# Super‐Heterostructures of Twisted Pd Nanoarrays Epitaxially Grown on Chiral Au Nanorods Boost Circularly Polarized Photocatalysis

**DOI:** 10.1002/advs.202502848

**Published:** 2025-04-24

**Authors:** Taotao Luo, Haoyu Li, Zhicheng Zhang, Shenli Wang, Xiaobin Pan, Stefanos Mourdikoudis, Chao Xue, Junjun Li, Kwok‐Yin Wong, Guangchao Zheng

**Affiliations:** ^1^ Colloidal Physics Group Key Laboratory of Materials Physics Ministry of Education School of Physics and Laboratory of Zhongyuan Light Zhengzhou University Zhengzhou 450001 P. R. China; ^2^ Institute of Quantum Materials and Physics Henan Academy of Sciences Zhengzhou 450046 P. R. China; ^3^ Key Laboratory of Organic Integrated Circuit, Ministry of Education & Tianjin Key Laboratory of Molecular Optoelectronic Sciences Department of Chemistry School of Science Tianjin University Tianjin 300072 P. R. China; ^4^ College of Food Science and Technology Henan University of Technology Zhengzhou 450001 P. R. China; ^5^ CINBIO Universidade de Vigo Department of Physical Chemistry Campus Universitario Lagoas Marcosende Vigo 36310 Spain; ^6^ State Centre for International Cooperation on Designer Low‐carbon and Environmental Materials School of Materials Science and Engineering Zhengzhou University Zhengzhou 450001 P. R. China; ^7^ State Key Laboratory of Chemical Biology and Drug Discovery Department of Applied Biology and Chemical Technology The Hong Kong Polytechnic University Hung Hom, Kowloon Hong Kong P. R. China

**Keywords:** chirality, circularly polarized photocatalysis, super‐heterostructres, surface plasmon resonance, twisted nanoarrays

## Abstract

Seed‐mediated growth provides an efficient way to produce chiral hybrid nanocrystals, mainly benefitting from the recent successful synthesis of discrete chiral metal nanocrystals. However, the chiroptical response of chiral core‐shell nanostructures decreases rapidly when the functional layer is grown on the discrete chiral metal nanocrystals. Herein, a synthetic methodology is demonstrated for the formation of chiral Au@Pd super‐heterostructures, in which Pd nanoarrays are epitaxially and densely grown with a twisted lattice on discrete chiral Au nanorods (Pd‐on‐cAuNRs). The highly aligned Pd nanoarrays on cAuNRs exhibit good chiroptical performance. Importantly, the Pd‐on‐cAuNR super‐heterostructures display chiral behavior in their catalytic actions, with the catalytic performance of D‐super‐heterostructures being twice higher than that of its L‐ super‐heterostructures. In addition, under circularly polarized light (CPL) irradiation, the chiral electromagnetic field and hot electrons generated on the chiral super‐heterostructures boost circularly polarized photocatalytic activity, the mechanism of which is supported by theoretical simulations and chiral photocurrent experiments. This research work offers fundamental insights on chiral surface plasmon resonance‐induced photocatalysis and rational design of chiral nanostructure photocatalysts.

## Graphical abstract

Au‐Pd super‐heterostructures not only retard the damping of chiroptical response but also enantio‐selectively enhance photocatalysis, thanks to the chiral electromagnetic field and chiral hot electrons.

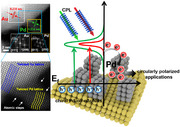

## 1 Introduction

Plasmon‐enhanced photocatalysis relies on plasmonic nanostructure catalysts which enable the coupling of photons and conduction electrons in plasmonic metallic nanoparticles (NPs).^[^ ^1^, ^2^, ^3^, ^4^, ^5^, ^6^ ^]^ The enhanced electromagnetic field and energetic hot electron‐hole carriers in the vicinity of plasmonic metal NPs have been demonstrated to play essential roles in plasmon‐enhanced photocatalysis. The hot electrons transferred into nearby active sites can activate the chemical energy and decrease the reaction energy barrier of reactant substrates.^[^ ^7^, ^8^, ^9^, ^10^, ^11^ ^]^ However, hot electrons are prone to recombine with holes, hindering the charge transfer and lowering the lifetime of charges, leading to low photocatalytic efficiencies.^[^ ^12^, ^13^, ^14^ ^]^ Therefore, the efficient use of hot electrons is an urgent issue to be addressed.

Recently, the development of chiral plasmonic photocatalysts with chiroptical response has provided a possible remedy for this challenge.^[^ ^15^, ^16^, ^17^, ^18^, ^19^, ^20^, ^21^ ^]^ The hot electrons generated on the chiral plasmonic nanostructures exhibit a chiral asymmetric behavior. When the CPL matches with the handedness of chiral nanomaterials, a higher catalytic performance was observed in comparison to that excited with the CPL of opposite polarization.^[^ ^22^, ^23^, ^24^, ^25^ ^]^ The chiral electromagnetic field and hot electrons can also improve the photocatalytic reaction. For instance, the photocatalytic efficiency of chiral Au@Ag hybrid core‐shell nanostructures is ca. 17 times higher than their analogous achiral nanostructures and it was also shown to be circularly polarized light‐dependent.^[^ ^26^ ^]^ It is worthy to note that plasmonic hybrid NPs composed of plasmonic metal NPs and other metal or semiconductor NPs can possess additional desirable properties on top of surface plasmon resonance (SPR). Especially, the additional nanodomains on hybrid NPs can improve the charge separation efficiency, extending the lifetime of hot electrons and functionalities. As an example, the reaction rate of chiral SiO_2_/Au NPs/TiO_2_ hybrid nanostructures for dye degradation and chiral Au@mesoporous SiO_2_ toward the oxidation of chiral 3,4‐dihydroxy‐phenylalanine is circular polarization‐dependent.^[^ ^27^, ^28^ ^]^ The generation of strong local electromagnetic field and high density of hot electrons occur when the vector of the excited CPL is parallel to the main axis of the chiral nanomaterials. Therefore, the development of chiral plasmonic hybrid NPs can address the challenges of plasmon‐enhanced photocatalysis.

Although a great progress in the shape‐control synthesis of chiral hybrids has been achieved, additional nanodomains on chiral metal NPs usually decrease their chiroptical response by several folds, particularly for core‐shell nanostructures. For instance, a Pd shell with few nanometer scale thickness on chiral Au helicoid NP leads to a nearly 20‐fold decrease of the anisotropic factor.^[^ ^29^ ^]^ This hybridization severely shields the original SPR absorption and the local electromagnetic field, resulting in a low efficiency of hot electron generation and transfer. On the other hand, Au─Pd heteromeric nanostructures not only allow the active sites to interact with an electron sacrificial agent, but also weaken the damping of SPR.^[^ ^30^ ^]^ In particular, Au─Pd or Au‐Rh super‐heterostructures in the form of Pd or Ir nanoarrays grown on Au nanoparticles display long lifetime of hot electrons in comparison to their core‐shell counterparts, thereby improving the photocatalytic efficiency.^[^ ^31^ ^]^ Although a lot of work has been done on the design of chiral metal nano‐catalysts, palladium nano‐arrays grown on discrete chiral gold nanoparticles with twisted lattices have not been reported so far in chiral plasmon‐enhanced photocatalysis.^[^ ^29^, ^32^, ^33^ ^]^

Herein, we propose a synthetic strategy to prepare chiral Au─Pd super‐heterostructures by directly growing an ordered Pd nanoarray on discrete chiral Au nanorod (cAuNR). In comparison to core‐shell nanostructures, these Pd nanoarrays grown on discrete chiral Au nanorods (Pd‐on‐cAuNRs) super‐heterostructures show only weak shielding effect toward the SPR of c‐Au NRs, maintaining their chiroptical response. Thus, Pd‐on‐cAuNRs display obvious chirality‐dependent behavior toward the catalytic reaction under the excitation of circularly polarized light. In addition, the unique structures of Pd‐on‐cAuNRs strengthen their photocatalytic performance thanks to the extended lifetime of hot electrons and unshielded plasmonic metal surface.

## 2 Results and Discussion

Initially we prepared discrete cAuNRs following a protocol developed in our laboratory.^[^ ^34^, ^35^ ^]^ The rod‐like morphologies of L‐cAuNRs and D‐cAuNRs were confirmed by TEM as depicted in Figure S1a,b (Supporting Information), with the longitudinal length and transverse width being ca. 200 and 100 nm respectively. The plasmonic CD (PCD) spectra of L‐cAuNRs and D‐cAuNRs at 621 nm are mirror‐like, according to their extinction spectra along the longitudinal and transverse axis (Figure S1c,d, Supporting Information). Following a reported protocol,^[^ ^31^ ^]^ Pd nanoarrays were grown on the cAuNRs via a seed‐mediated growth approach. The overgrowth of Pd adatoms was carefully controlled by the concentrations of cetylpyridinium chloride monohydrate (CPC), the reducing agent (ascorbic acid, AA), and the Pd precursors (K_2_PdCl_4_).

**Figure 1:**
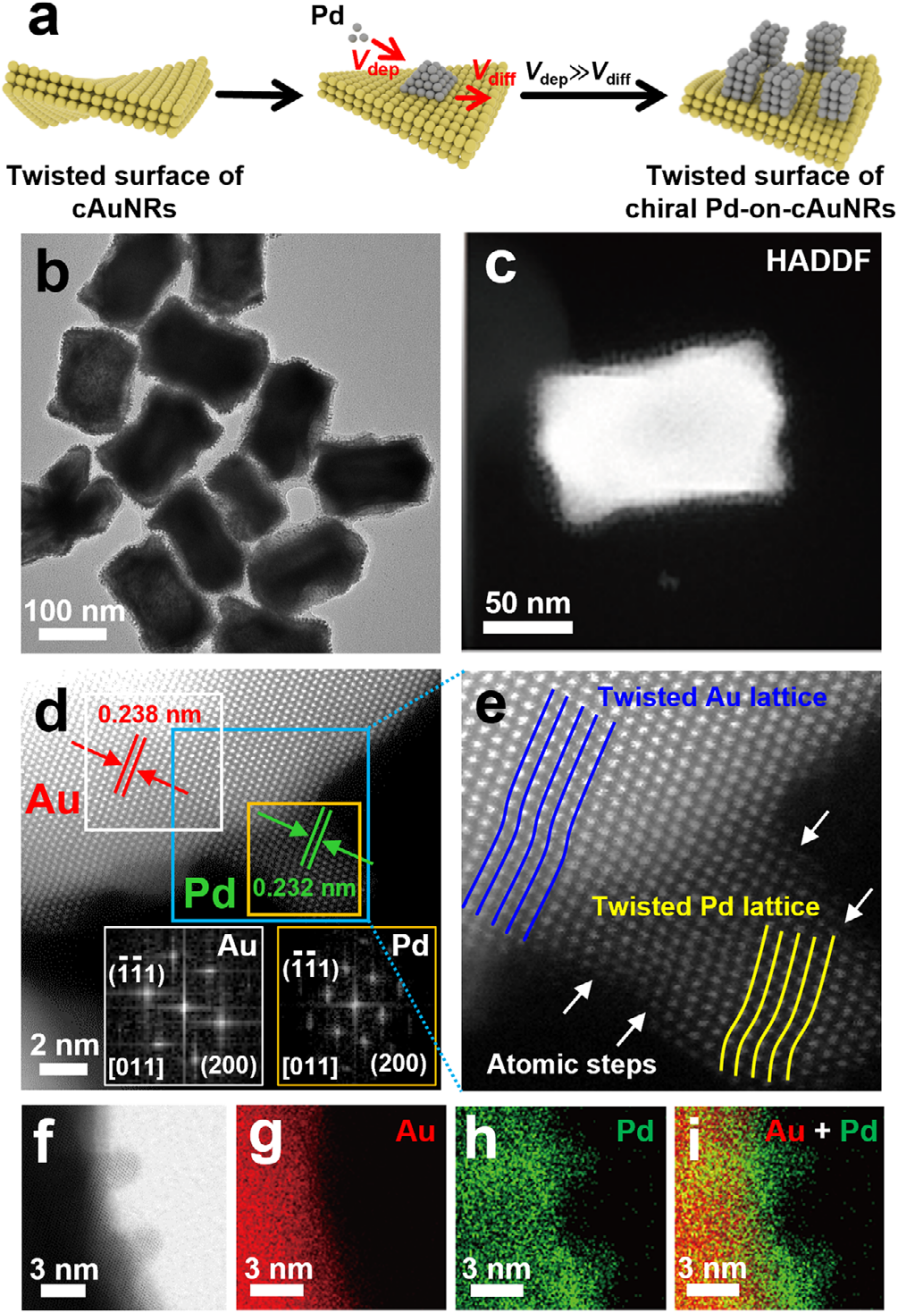
a) Schematic diagram of seed‐mediated growth for chiral Pd‐on‐cAuNRs super‐heterostructure based on discrete cAuNRs. b) TEM image of L‐Pd‐on‐cAuNRs. c) HAADF‐STEM image of L‐Pd‐on‐cAuNRs. d) Spherical aberration‐corrected HAADF‐STEM image of a selected area on a L‐Pd‐on‐cAuNRs. Insets are the corresponding FFT patterns of the selected area marked in (d). e) Enlarged spherical aberration‐corrected HAADF‐STEM image taken from the blue dashed rectangle in (d). f) Spherical aberration‐corrected STEM image of a selected area on a L‐Pd‐on‐cAuNRs. g–i) corresponding EDS elemental mapping of the Au core (g), Pd nanoarrays (h), and overlapped (i) images of the L‐Pd‐on‐cAuNRs in (f).

When the concentration of reducing agent and Pd precursor were relatively low, Pd nanoarrays on cAuNRs with a small size and sparse density were formed, indicating that the deposition rate was low due to the limited extent of the reduction of Pd ions (Figure S2a,b, Supporting Information). In contrast, when a higher amount of reducing agent and Pd precursor were employed, Pd nanoarrays were grown on cAuNRs, while a small amount of Pd nanodots also appeared in solution, which indicated that self‐nucleation of Pd ions occurred because the reduction rate is faster than the deposition rate (V_dep_) (Figure S2c,d, Supporting Information). Only when the deposition rate is higher than the diffusion rate (V_diff_), Pd nanoarrays could grow along the <111> direction. As a result, the growth of Pd atoms follows a Volmer‐Weber mode, resulting in the growth of Pd nanoarrays on discrete chiral Au nanorods (Pd‐on‐cAuNRs) in a high‐density manner (**Figure** **1**a). The TEM images of the Pd‐on‐cAuNRs are shown in Figure 1b and Figure S3 (Supporting Information), using L‐cAuNRs and D‐cAuNRs seeds, respectively. The diameter and length of the Pd nanoarrays were measured to be ≈6.0 ± 0.9 and 3.9 ± 0.9 nm, respectively (Figure S4, Supporting Information). High‐density Pd nanoarrays with a darker contrast grew vertically on the surface of cAuNRs which showed a brighter contrast, as revealed in the high‐angle dark‐field scanning TEM (HAADF‐STEM) images (Figure 1c). Besides, the Pd nanoarrays align mostly in a row. From the spherical aberration‐corrected HAADF‐STEM image and using FFT analysis, the lattice fringes were measured to be ≈0.238 and 0.232 nm, which can be ascribed to the ($\bar{1}\bar{1}1$) and (200) plane of Au and Pd, respectively (Figure 1d). In addition, the lattices of Pd nanoarrays are significantly twisted due to its epitaxial growth on the cAuNR cores, as confirmed by the spherical aberration‐corrected HAADF‐STEM image. The intrinsic chirality of cAuNRs also displayed a twisted chiral lattice (Figure 1e). The twisted chiral lattices of Pd were contributed by the chirality transfer of intrinsic chirality of cAuNRs or by the macroscopic shear forces.^[^ ^36^ ^]^ Elemental (EDX) mapping of selected Pd‐on‐cAuNRs clearly demonstrated that cAuNRs are centered at the core and Pd nanoarrays are distributed around cAuNRs, illustrating that the Pd nanoarrays were successfully grown on the surface of cAuNRs (Figure 1f–i).

**Figure 2:**
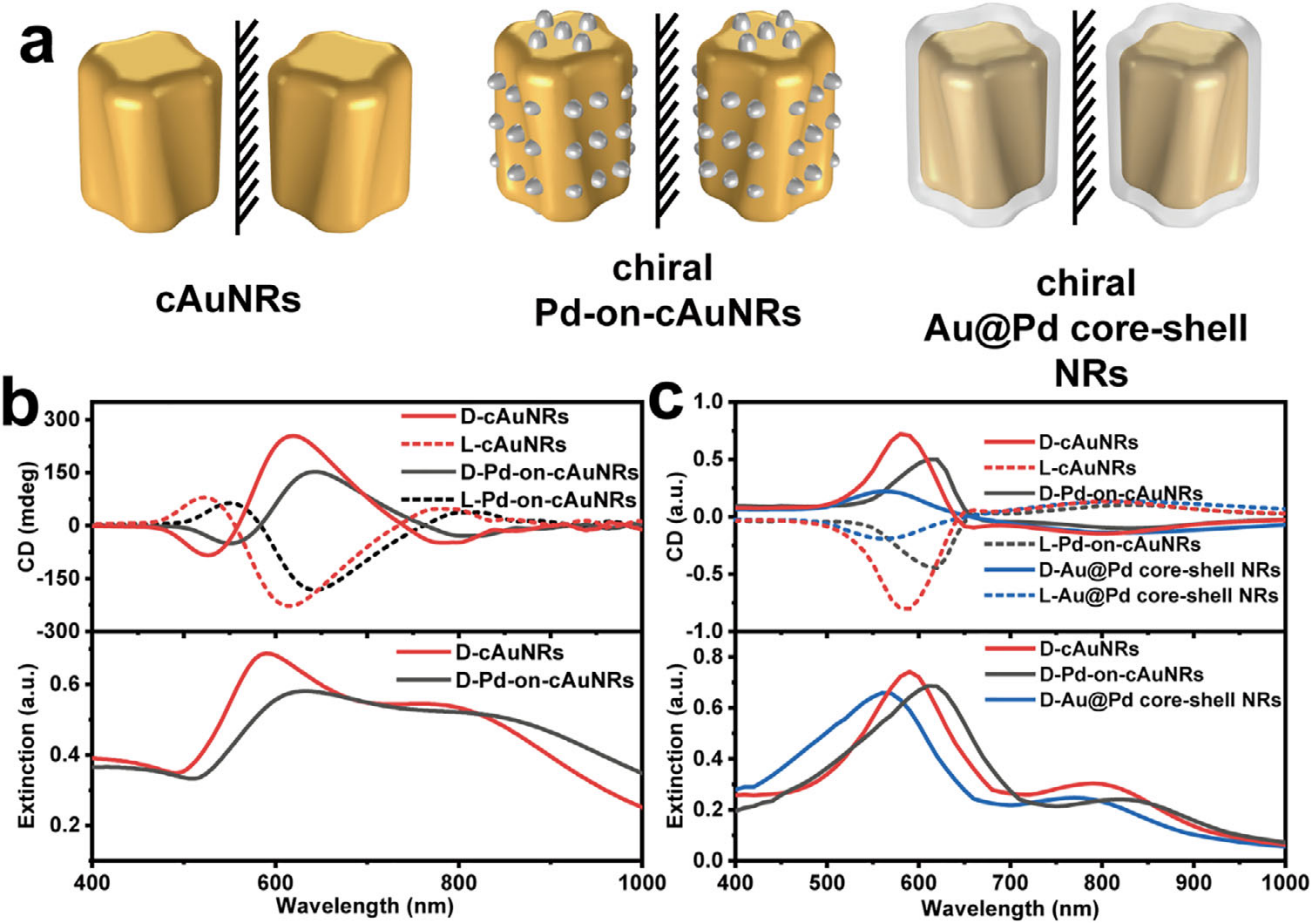
a) Schematic illustrations of chiral Pd‐on‐cAuNRs and chiral Au@Pd core‐shell NRs model. b) Experimental extinction (bottom) and PCD spectra (top) of chiral Pd‐on‐cAuNRs. c) Simulated extinction (bottom) and PCD spectra (top) of chiral Pd‐on‐cAuNRs model and chiral Au@Pd core‐shell NRs.

Accordingly, we have built the morphology model for cAuNRs, Pd‐on‐cAuNRs super‐heterostructures, and chiral Au@Pd core‐shell NRs (**Figure** **2**a). The PCD spectra of Pd‐on‐cAuNRs super‐heterostructures experience a red‐shift to 642 and 823 nm, while a red‐shift of TSPR bands from 585 to 648 nm and LSPR bands from 786 to 938 nm, respectively, were also observed (Figure 2b). In contrast to the silent chiroptical response of pure chiral Pd helicoids, Pd‐on‐cAuNRs super‐heterostructures features a stronger g‐factor of 0.01.^[^ ^37^ ^]^

To examine the distinct chiroptical response of chiral Pd‐on‐cAuNRs structures, we used COMSOL Multiphysics commercial software to conduct simulations concerning the electromagnetic behavior of these materials. The diameter, length, and pitch depth of cAuNR were set to 50, 140, and 10 nm to match the synthesized structure, respectively. The length of Pd nanoarray was set to 20 nm and the width to 12 nm (Figure 2c). The simulation region is a cube with a side length of 700 nm, surrounded by an 80 nm‐thick perfectly matched layer (PML) used to absorb the light propagated to the boundary to simulate infinite space. We use the dielectric function for Au from Johnson‐Christy's experiment, and Pd from the experiment of Windt et al.^[^ ^38^, ^39^ ^]^ The electric field on the structure surface is obtained by solving Maxwell's equations. The incident field propagates along the x‐direction, and the background refractive index is adjusted to 1.33. We first give the absorption and CD spectra of cAuNRs, chiral Au@Pd core‐shell NRs, and chiral Pd‐on‐cAuNRs in Figure 1j. It can be seen that there are two distinct characteristic peaks in the extinction and CD spectra corresponding to the longitudinal and transverse plasmon modes respectively. The extinction and CD peaks of chiral Pd‐on‐cAuNRs are red‐shifted relative to cAuNRs. However, the absorption and CD peaks of the chiral Au@Pd core‐shell NRs are blue‐shifted compared to the cAuNRs. If a shell is coated on a metallic nanocrystal, the real part of its dielectric function should be written as in Equation (1):

(1)

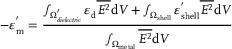

where Ω_shell_ is the volume of the shell layer, and 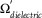 is the volume of the surrounding medium excluding the shell.^[^ ^40^ ^]^ If the continuous shell is Pd, the first term on the right side of the equation is 0, and since the real part of the dielectric function of the metal is negative, the reduction of the real part of the dielectric function of the structure results in a blue shift in the characteristic peak in the spectra. In addition, an increase in the thickness of the continuous shell leads to a larger aspect ratio, which also results in blue shift of the spectra. When the structure is chiral Pd‐on‐cAuNR, due to discontinuous shell of Pd particles and the surrounding medium being mostly water, the real part of dielectric function of the structure increases and the characteristic peak is red‐shifted. The poor optical response of chiral Au─Pd hybrids is contributed by the large imaginary part of the Pd dielectric function.^[^ ^41^ ^]^

To further study and validate the growth procedure and formation of the super‐heterostructures, we investigated the morphology evolution during different growth stages. Pd nanoarrays became detectable in a very short time (about one minute) when the reducing agent was injected into the growth solution. The length and width of Pd nanoarrays gradually increased while its density also become more visible in a few minutes (Figure S5a–c, Supporting Information). Meanwhile, the extinction spectra and PCD spectra were red‐shifted (Figure S5d, Supporting Information). In contrast, the chiral Au@Pd core‐shell NRs showed a very weak chiroptical response due to plasmon damping by Pd shells which totally covered the cAuNRs (Figure S6, Supporting Information). The surface of cAuNRs was not fully covered by the Pd nanoarrays in Pd‐on‐cAuNRs, leading to a good chiroptical response for the Pd‐on‐cAuNRs.

**Figure 3:**
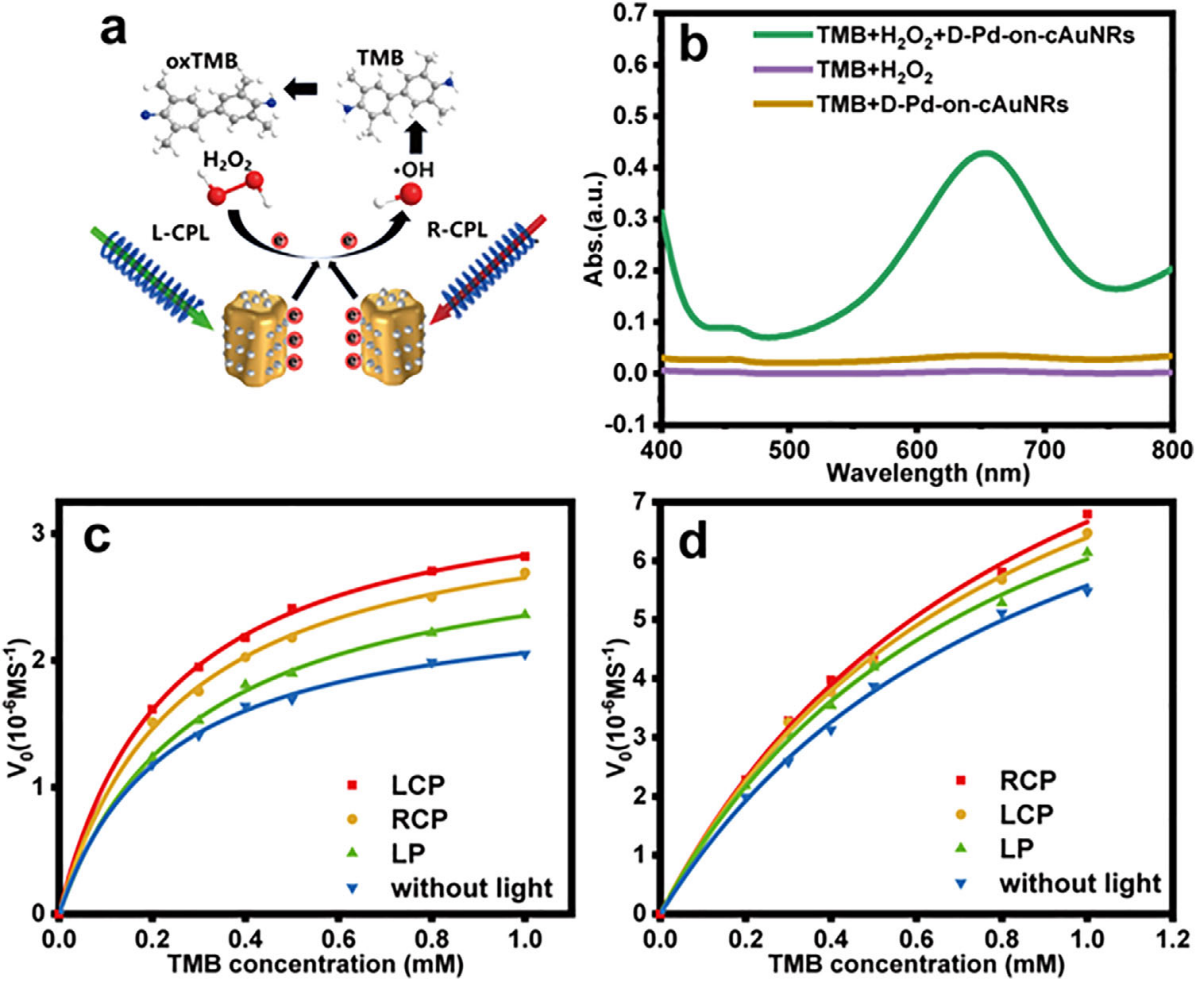
a) Schematic diagram of TMB oxidation by H_2_O_2_ in the presence of cAuNRs. b) POD‐like activity analysis of chiral Pd‐on‐cAuNRs. c,d) The reaction rate of L‐Pd‐on‐cAuNRs and D‐Pd‐on‐cAuNRs with varying TMB concentration under different light illumination conditions, respectively.

In order to investigate the circular polarization‐sensitive photocatalysis of chiral Pd‐on‐cAuNRs heterostructures, we explored their photocatalytic performance enhanced by chiral plasmon toward nanozyme reactions and nitrophenol reduction reaction. Before investigating the nanozyme reaction, it is essential to systematically evaluate the influence of pH and temperature on the catalytic reaction rate of POD‐like nanozymes. Experimental results indicate that the catalytic reaction rate is maximized when the pH is maintained between 3.5 and 4.5 and the temperature is controlled at 35 °C, as illustrated in Figure S7 (Supporting Information). These findings establish the optimal conditions for achieving maximum catalytic performance in subsequent nanozyme reaction. First, the oxidation of 3,3ʹ,5,5ʹ ‐tetramethylbenzidine (TMB) in the presence of H_2_O_2_ was conducted using chiral Pd‐on‐cAuNRs heterostructures upon the excitations of left circularly polarized light (LCP), right circularly polarized light (RCP) and linearly polarized light (LP), respectively (**Figure** **3**a). The main absorption peak at ca. 652 nm was monitored with the reaction time, assigned to the formation of ox‐TMB. As shown in the Figure 3b, the reaction took place only when the reaction solution contained TMB, H_2_O_2_, and chiral Pd‐on‐cAuNRs heterostructures, demonstrating that these heterostructures display an enzyme‐mimicking catalytic activity. Figures S8–S11 (Supporting Information) show a gradual increase in ox‐TMB absorption peaks, which reached a plateau after a certain reaction time. Kinetic analysis for different [TMB] was conducted by tracking the time evolution of the absorption intensity at 652 nm. The initial reaction rates (V_0_) were derived from the primary linear segments of the curves, using the molar absorption coefficient for ox‐TMB. Figure 3c,d, and Figure S12 (Supporting Information) show the variation of V_0_ as a function of [TMB], as well as its fitting to the Michaelis‐Menten model. Figure S13 (Supporting Information) presents the variation of V_0_ as a function of [H_2_O_2_], along with its fitting to the Michaelis‐Menten model (Equation 2).

(2) $$V_{0}=\frac{V_{\max}\left[\mathrm{TMB}\right]}{K_{m}+\left[\mathrm{TMB}\right]}$$

where V_max_ represents the maximum reaction velocity achieved by an enzyme‐catalyzed reaction when the substrate concentration is saturating, and K_m_ is the Michaelis‐Menten constant which reflects the affinity of the Pd‐on‐cAuNR for the TMB.

From the fitting, under darkness, the K_m_ and V_max_ value of TMB were 0.82 mm and 9.97 × 10^−7^ Ms^−1^ for D‐Pd‐on‐cAuNR, respectively. Those values of H_2_O_2_ for D‐Pd‐on‐cAuNR were 207.46 mm and 10.8 × 10^−7^ Ms^−1^ correspondingly. For L‐Pd‐on‐cAuNR, the K_m_ and V_max_ value of TMB were 0.23 mm and 2.5 × 10^−7^ Ms^−1^ respectively, and those values of H_2_O_2_ were 196.2 mM and 6.3 × 10^−7^ Ms^−1^, respectively (see **Table** **1**). Additionally, we estimated the catalytic efficiency, k_eff_, which is typically utilized to describe the catalytic performance of an enzyme. k_eff_ is defined as k_cat_/K_m_, where k_cat_, the catalytic number, represents the ratio of V_max_ to the amount of nanozymes. This metric reflects the nanozyme's ability to catalyze a specific substrate (see k_eff_ values in Table 1). Finally, we calculated the selective factor, defined as the ratio of k_eff_ for L‐ and D‐Pd‐on‐cAuNRs, which indicates enantioselectivity toward enzymatic catalysis. Notably, during the oxidation of TMB in the presence of chiral Pd‐on‐cAuNRs under dark conditions, the selective factor for TMB as a substrate was estimated to be 0.89, and for H_2_O_2_ it was 0.62 (Table 1). This suggests that the catalytic efficiency of D‐Pd‐on‐cAuNRs is greater than that of L‐Pd‐on‐cAuNRs. Moreover, the POD‐like performance of chiral Pd‐on‐cAuNRs was examined under LCP and RCP light (Figures S8–S11, Supporting Information). Circular polarization‐dependent catalytic activities were observed for L‐Pd‐on‐cAuNRs and D‐Pd‐on‐cAuNRs (Table 1). We observed that when L‐Pd‐on‐cAuNRs were used as photocatalysts under LCP excitation, they exhibited a higher reaction rate than that under RCP excitation. Similarly, RCP displayed a higher reaction rate when it was applied for the illumination of the D‐Pd‐on‐cAuNRs, rather than the excitation of opposite LCP. Under the RCP conditions, the K_m_ and V_max_ value of TMB for D‐Pd‐on‐cAuNRs were 0.43 mm and 12.7 × 10^−7^ Ms^−1^, respectively, and those values of H_2_O_2_ were 248.8 mm and 20.3 × 10^−7^ Ms^−1^ correspondingly. For L‐Pd‐on‐cAuNR, the K_m_ and V_max_ value of TMB were 0.23 mm and 3.23 × 10^−7^ Ms^−1^. Those values for H_2_O_2_ were 198.4 mm and 7.9 × 10^−7^ Ms^−1^. The selective factor for TMB as a substrate was estimated to be 0.62, and for H_2_O_2_, it was 0.49. Under the LCP conditions, the K_m_ and V_max_ value of TMB for D‐Pd‐on‐cAuNRs were 0.79 mm and 11.5 × 10^−7^ Ms^−1^, respectively, and those values for H_2_O_2_ were 196.1 mm and 12.6 × 10^−7^ Ms^−1^. For L‐Pd‐on‐cAuNR, the K_m_ and V_max_ value of TMB were 0.24 mm and 3.4 × 10^−7^ Ms^−1^, respectively, and those values for H_2_O_2_ were 239.9 mm and 10.5 × 10^−7^ Ms^−1^. The selective factor for TMB as a substrate was estimated to be 0.97, and for H_2_O_2_, it was 0.67 (Table 1). In addition, the activity performance of chiral Pd‐on‐cAuNRs under the excitation of LP was between the values derived under excitation of RCP and LCP. The selective factor for TMB as a substrate was estimated to be 0.82, and for H_2_O_2_, it was 0.65. Notably, chiral Pd‐on‐cAuNRs heterostructures show obvious polarization‐dependent nanoenzyme reaction. The whole reaction was carried out in the water bath at a set temperature of 30 °C, canceling out the thermal effect induced by the phonon–phonon scattering. We conclude that CPL‐dependent photocatalysis could be related to circularly polarized hot‐electrons induced by the chiral plasmons.

**Table 1:** Comparison of the V_max_, K_m_, k_cat_, k_eff_, and selective factor for either TMB or H_2_O_2_ substrate using D‐Pd‐on‐cAuNRs and L‐Pd‐on‐cAuNRs as nanozymes in the absence (N.A.) and the presence of different polarized light illumination.

Light	Nanozymes	substrate	V_max_ [M s^−1^]	[K_m_][mM]	[K_cat_][Ms^−1^ g^−1^]	[K_cat_]/[K_m_] [s^−1^ g^−1^]	Selective factor
N.A.	L‐Pd‐on‐cAuNRs	TMB	2.5 × 10^−7^	0.23	4.5 × 10^−5^	2.0 × 10^−1^	0.89
		H_2_O_2_	6.3 × 10^−7^	196.2	1.1 × 10^−4^	5.8 × 10^−4^	
	D‐Pd‐on‐cAuNRs	TMB	10,0 × 10^−7^	0.82	1.8 × 10^−4^	2.2 × 10^−1^	0.62
		H_2_O_2_	10.8 × 10^−7^	207.5	2.0 × 10^−4^	9.4 × 10^−4^	
LP	L‐Pd‐on‐cAuNRs	TMB	3.2 × 10^−7^	0.28	5.8 × 10^−5^	2.1 × 10^−1^	0.82
		H_2_O_2_	6.4 × 10^−7^	166.0	1.2 × 10^−4^	7.0 × 10^−4^	
	D‐Pd‐on‐cAuNRs	TMB	10.6 × 10^−7^	0.77	1.9 × 10^−4^	2.5 × 10^−1^	0.65
		H_2_O_2_	11.5 × 10^−7^	194.2	2.1 × 10^−4^	1.1 × 10^−3^	
RCP	L‐Pd‐on‐cAuNRs	TMB	3.2 × 10^−7^	0.23	5.8 × 10^−5^	2.5 × 10^−1^	0.46
		H_2_O_2_	7.9 × 10^−7^	198.4	1.4 × 10^−4^	7.2 × 10^−4^	
	D‐Pd‐on‐cAuNRs	TMB	12.7 × 10^−7^	0.43	2.3 × 10^−4^	5.4 × 10^−1^	0.49
		H_2_O_2_	20.3 × 10^−7^	248.8	3.7 × 10^−4^	1.5 × 10^−3^	
LCP	L‐Pd‐on‐cAuNRs	TMB	3.4 × 10^−7^	0.24	6.2 × 10^−5^	2.6 × 10^−4^	0.97
		H_2_O_2_	10.5 × 10^−7^	239.9	1.9 × 10^−4^	7.9 × 10^−7^	
	D‐Pd‐on‐cAuNRs	TMB	11.5 × 10^−7^	0.79	2.1 × 10^−4^	2.6 × 10^−4^	0.67
		H_2_O_2_	12.6 × 10^−7^	196.1	2.3 × 10^−4^	1.2 × 10^−6^	

**Figure 4:**
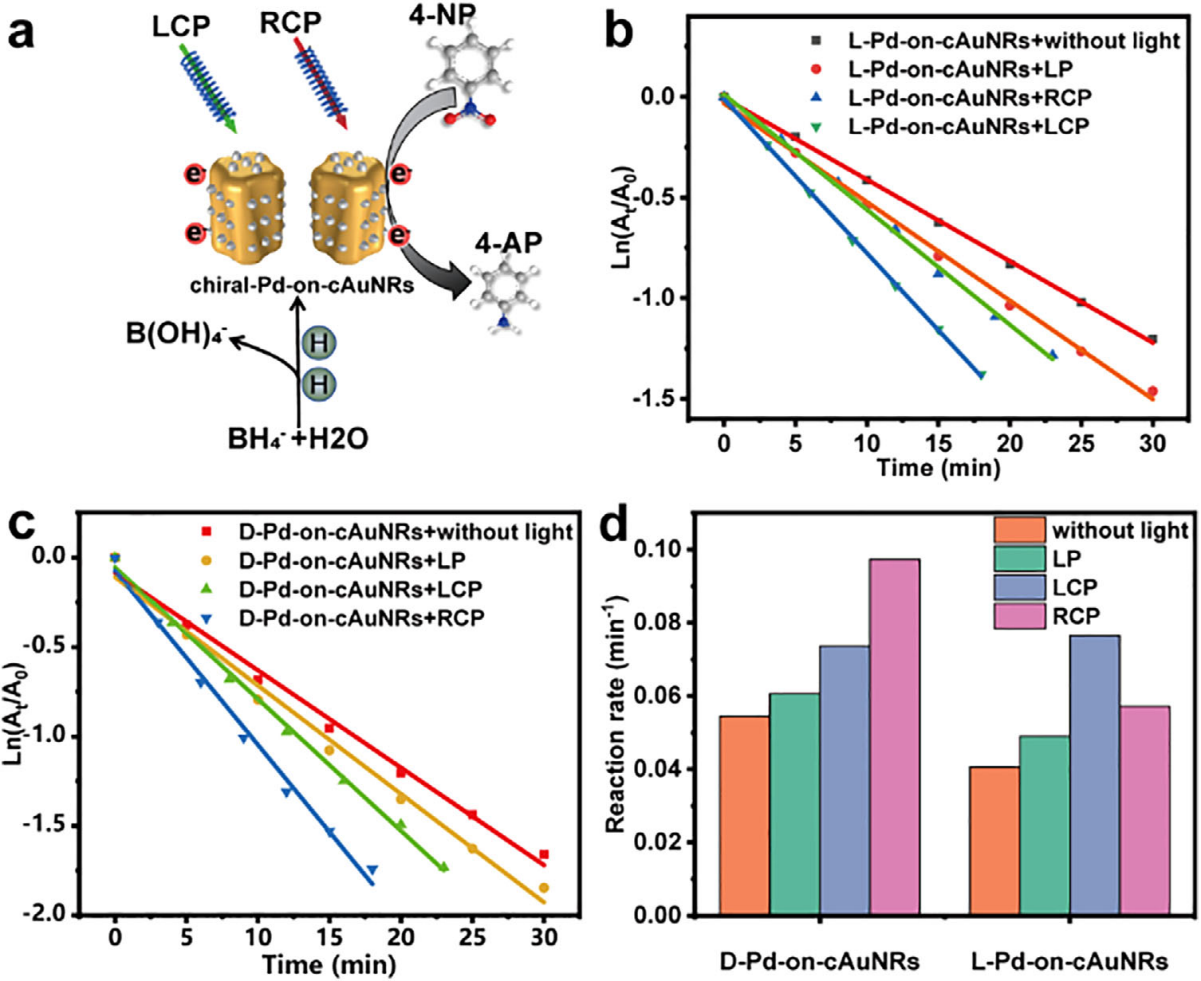
a) Schematics of chiral Pd‐on‐cAuNRs photocatalysts involved in the nitro‐reduction process. b,c) Plot of Ln (A_t_/A_0_) as a function of time for the reaction catalyzed by L‐Pd‐on‐cAuNRs and D‐Pd‐on‐cAuNRs under different polarized light excitations, respectively. d) Histograms for the comparison of reaction rate under different polarized light excitations.

Besides, the circular polarization‐sensitive catalytic activities of the chiral Pd‐on‐cAuNRs were also evaluated for the reduction of 4‐nitrophenol (4‐NP) to 4‐aminophenol (4‐AP) via BH_4_^−^. During this model reaction, a gradual disappearance of the yellow color of the reaction solution takes place under the irradiation of different polarized lights (LP, LCP, and RCP, see **Figure** **4**a). This reaction can be typically monitored by time‐resolved UV–vis–NIR spectroscopy, since the characterized absorption peak of nitrophenol ions and 4‐AP at ca. 400 nm are known to decrease in the course of the reaction (Figures S14 and S15, Supporting Information).^[^ ^42^ ^]^ By analyzing the reaction kinetics, the reaction rate of this photocatalytic reaction is calculated from the equation ln (*A_t_*/*A*_0_) = *K_t_*  where A_t_ and A_0_ represent the absorption intensity at wavelength of 400 nm in the beginning and at reaction time t, respectively. *K_t_* obtained from the pseudo‐first‐order kinetics equation represent its reaction rate (Figure 4b,c). Although the reaction takes place under dark conditions, both of the reduction efficiency and reaction rates were lower in comparison to that under the irradiation of light. Furthermore, irradiation with LP yielded a low reaction rate and catalytic efficiency compared to the irradiation with RCP and LCP. For L‐Pd‐on‐cAuNRs, there is a higher catalytic efficiency and reaction rate under the illumination of LCP than under RCP. A similar trend was observed for D‐Pd‐on‐cAuNRs: a higher catalytic efficiency and reaction rate was evidenced under the illumination of RCP in comparison to that under LCP (Figure 4d). The reaction rates of D‐Pd‐on‐cAuNRs under different light conditions were 0.097, 0.074, 0.061, and 0.054 min^−1^, the reaction rates of L‐Pd‐on‐cAuNRs under different light conditions were 0.077, 0.057, 0.049, and 0.041 min^−1^.

This further illustrates that aligning the κ‐vector of the excited CPL with the helical axis of chiral nanomaterials can significantly enhance the chiral plasmonic effect, thereby improving the reaction rate and photocatalytic efficiency.

**Figure 5:**
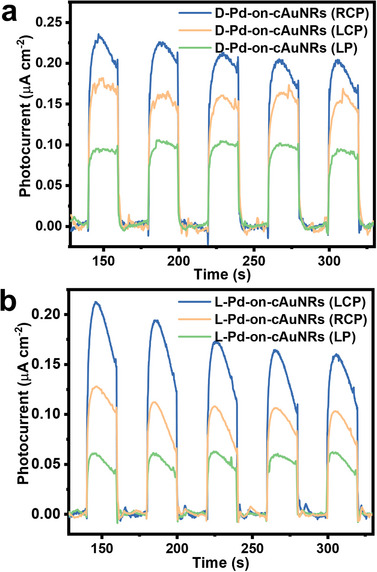
a) Time‐dependent photocurrents of the D‐Pd‐on‐cAuNRs under different polarized light excitations, respectively. b) Time‐dependent photocurrents of the L‐Pd‐on‐cAuNRs under different polarized light excitations, respectively.

Moreover, the understanding of chiral hot electron‐enhanced photocatalysis provides fundamental insight into chiral plasmon‐enhanced photocatalytic applications. We investigated the hot electron generation in the chiral Pd‐on‐cAuNRs using an electrochemical workstation. The particle densities of L‐Pd‐on‐cAuNRs and D‐Pd‐on‐cAuNRs on the glassy carbon electrode were carefully adjusted to be appropriately equal. As shown in **Figure** **5**, the hot electron photocurrent contributed by D‐Pd‐on‐cAuNR is 0.022 µA cm^−2^ under RCP light, which is 1.5‐fold higher than that of LCP light environment and is 2.2‐fold higher than that of LP light environment. But the hot electron photocurrent contributed by L‐Pd‐on‐cAuNR is 0.019 µA cm^−2^ under LCP light, which is 1.35‐fold higher than that of RCP light environment and is 2.7‐fold higher compared to that of LP light environment. The polarized light‐dependent improvement in photocurrent suggests that the enantio‐selective interaction between chiral Pd‐on‐cAuNRs and circularly polarized light facilitate hot electrons generation, which triggers the chiral plasmon‐enhanced photocatalytic reaction.

The chirality of the system can be represented by **C**_**E**_. For a monochromatic source traveling with angular frequency **ω**, the optical chiral density as a function of the position is given by Equations ((3), (4), (5)).

(3) $$C_{E}=\left|\frac{C}{C_{\mathrm{CPL}}}\right|$$

(4) C=−ε0ω2ImE∗·B

(5) $$C_{\mathrm{CPL}}^{\pm }=\pm \frac{\varepsilon_{0}\omega }{2c}{\left|E\right|}^{2}$$

where C_CPL_ is the optical chiral density for circularly polarized light with orthogonal field vectors, equal magnitudes, and a ±π/2 phase. The parameter C_E_ represents the distortion of electromagnetic field compared with circularly polarized light. As the increase of Pd thickness, C_E_ increases from 0.28 to 0.94, which is consistent with the changing trend of CD (Figure S16, Supporting Information). However, with the same thickness of Pd shell, chiral Pd‐on‐cAuNRs display a higher in comparison to chiral Au@Pd core‐shell NRs.

**Figure 6:**
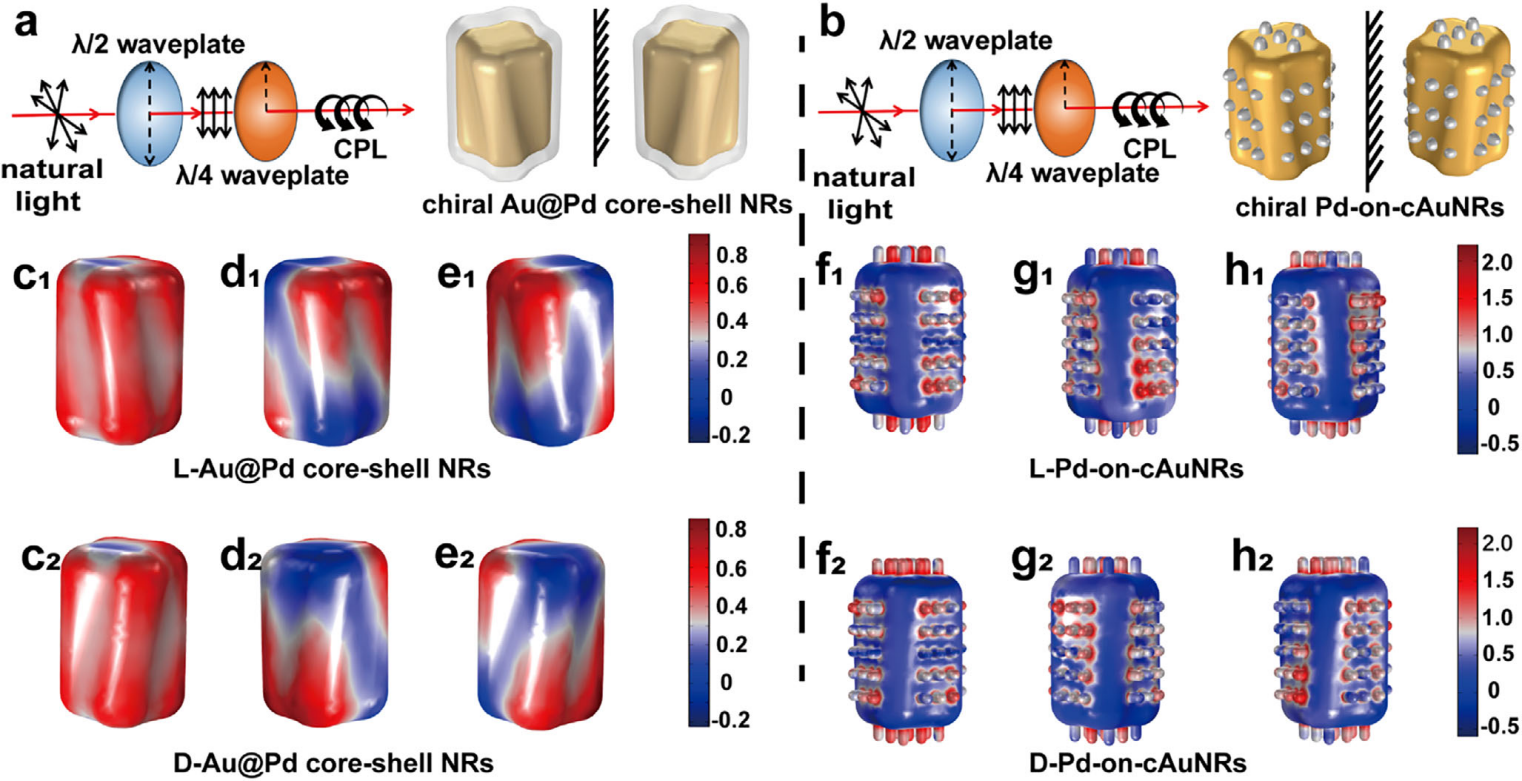
a) Schematics of chiral Au@Pd core‐shell NRs under different polarized light illumination. b) Schematics of chiral Pd‐on‐cAuNRs under different polarized light illumination. (c_1_ and c_2_) Electromagnetic simulation of L‐Au@Pd core‐shell NRs and D‐Au@Pd core‐shell NRs under LP illumination. (d_1_ and d_2_) Electromagnetic simulation of L‐Au@Pd core‐shell NRs and D‐Au@Pd core‐shell NRs under LCP illumination. (e_1_ and e_2_) Electromagnetic simulation of L‐Au@Pd core‐shell NRs and D‐Au@Pd core‐shell NRs under LCP illumination. f_1_ and f_2_) Electromagnetic simulation of L‐Pd‐on‐cAuNRs and D‐Pd‐on‐cAuNRs under LP illumination. g_1_ and g_2_) Electromagnetic simulation of L‐Pd‐on‐cAuNRs and D‐Pd‐on‐cAuNRs under LCP illumination. (h_1_ and h_2_) Electromagnetic simulation of L‐Pd‐on‐cAuNRs and D‐Pd‐on‐cAuNRs under RCP illumination.

The simulated electric field enhancement distributions of the chiral Au@Pd core‐shell NRs and chiral Pd‐on‐cAuNRs are depicted in **Figure** **6**, where E_0_ is the intensity of incident field. It can be observed that the distribution of electric field enhancement under LP light illumination is uniform, and the maximum value is ≈0.68. However, the electric field enhancement distribution of chiral Au@Pd core‐shell NRs by CP light illumination is mainly controlled by the chirality of incident light, and its maximum value can exceed 0.8, which is higher than that of LP light. Therefore, CPL promotes the electric field enhancement of chiral Pd‐on‐cAuNRs. For the chiral Pd‐on‐cAuNRs structure, the electric field enhancement of both LP and CP light are stronger than that of chiral Au@Pd core‐shell NRs. Moreover, the electric field enhancement is mainly distributed at the interface between the Pd nanoarray and the cAuNRs, indicating that the Pd nanoarray enhances the chiral electromagnetic field of the structure, which in turn affects the catalytic efficiency. In addition, with the increase of Pd shell thickness, the electric field enhancement decreases gradually. The observed phenomenon can be elucidated by the fact that Pd possesses a reduced number of free electrons compared to Au, which consequently results in a diminished plasmonic effect.

In summary, by manipulating the ratio between deposition rate and diffusion rate through the adjustment of thermodynamic parameters including surfactants, reducing agents, and precursor concentration, super‐heterostructures of chiral Pd‐on‐cAuNRs were successfully produced, demonstrating an excellent chiroptical response for hybrid nanostructures. The catalytic activity of chiral Pd‐on‐cAuNRs displays a chiral plasmon‐enhanced photocatalytic performance, that is, an enhanced photocatalytic reaction toward the nanozyme reaction and nitrophenol reduction reaction under the excitation of circularly polarized light compared to that under linearly polarized light and dark conditions. In addition, the reaction rate of chiral Pd‐on‐cAuNRs is higher when it is excited under light conditions with the same conformation than that under light conditions with the opposite conformation, which is corroborated by theoretical simulations. Thus, this synthetic strategy yielding chiral super‐heterostructures provides a promising way for the construction of chiral hybrid photocatalysts. Meanwhile, the circular polarization‐dependent photocatalytic performance due to the asymmetric electromagnetic field and hot electrons shows a great potential for the chiral plasmon‐induced circularly polarized photocatalysis.

## Conflict of Interest

The authors declare no conflict of interest.

## Supporting information



Supporting Information

## Data Availability

The data that support the findings of this study are available from the corresponding author upon reasonable request.
