# Supplementary material for: Super‐Heterostructures of Twisted Pd Nanoarrays Epitaxially Grown on Chiral Au Nanorods Boost Circularly Polarized Photocatalysis
Source: Adv Sci (Weinh). 2025 Apr 24;12(26):2502848. doi: 10.1002/advs.202502848 (PMC12245117; doi:10.1002/advs.202502848)
Supplement: Supplementary file 1 — Supporting Information [file ADVS-12-2502848-s001.docx]

Supporting Information

Super-heterostructures of Twisted Pd Nanoarrays Epitaxially Grown on Chiral Au Nanorods Boost Circularly Polarized Photocatalysis

Taotao Luo,^a,b#^ Haoyu Li^a,b#^ Zhicheng Zhang,^c#^ Shenli Wang^d*^ Xiaobin Pan^a,b^, Stefanos Mourdikoudis^e^, Chao Xue^f^, Junjun Li^c^, Kwok-Yin Wong,^g*^ Guangchao Zheng^a,b*^

^a^ Colloidal Physics Group, Key Laboratory of Materials Physics, Ministry of Education, School of Physics and Laboratory of Zhongyuan Light, Zhengzhou University, Zhengzhou 450001, P. R. China.

^b^ Institute of Quantum Materials and Physics, Henan Academy of Sciences, Zhengzhou 450046, P. R. China.

^c^ Key Laboratory of Organic Integrated Circuit, Ministry of Education & Tianjin Key Laboratory of Molecular Optoelectronic Sciences, Department of Chemistry, School of Science, Tianjin University, Tianjin 300072, P. R. China

^d^ College of Food Science and Technology, Henan University of Technology, Zhengzhou 450001, P. R. China.

^e^ CINBIO, Universidade de Vigo, Department of Physical Chemistry, Campus Universitario Lagoas Marcosende, 36310 Vigo, Spain.

^f^ State Centre for International Cooperation on Designer Low-carbon and Environmental Materials, School of Materials Science and Engineering, Zhengzhou University, Zhengzhou, 450001 China

^g^ State Key Laboratory of Chemical Biology and Drug Discovery, Department of Applied Biology and Chemical Technology, The Hong Kong Polytechnic University, Hung Hom, Kowloon, Hong Kong (China).

**Materials:** Tetrachloroauric acid hydrated (HAuCl_4_·4H_2_O, analytical grade), sodium tetrachloropalladate (Na_2_PdCl_4_, 98%), silver nitrate (AgNO_3_, 99%), hexadecylpyridinium chloride monohydrate (CPC, 99-102%), hexadecyltrimethylammonium bromide (CTAB, >98%), hexadecyltrimethylammonium chloride (CTAC, > 98%), sodium oleate (NaOL, > 97%), ascorbic acid (AA, 99%) and cysteine hydrochloride monohydrate (L-Cys, D-Cys, >99%), sodium borohydride (NaBH_4_, 98%) were purchased from Adamas. HCl (37 wt % in water, 12.1 M) was bought from Fisher Scientific. 3’3’5’5-Tetramethylbenzidine hydrochloride (TMB), 4-nitrophenol (4-NP) and hydrogen peroxide (H_2_O_2_) were supplied from Macklin. The deionized water used in our experiments was obtained from the Milli-Q System. All the chemicals were used as received without further purification.

**Synthesis of Au nanorods (NRs):** The production of AuNRs was carried out by following reported seed-mediated growth approaches. ^[1]^ Initially, 10 mL of 0.5 mM HAuCl_4_ was injected into 10 mL of 0.2 M CTAB solution, and the mixture was shaken for a while. Then, 2 mL of 6 mM NaBH_4_ were added into the above solution under vigorous stirring for 2 min. The obtained seed solution was kept at room temperature for a few minutes. To prepare the growth solution, 3.6 mL of 4 mM AgNO_3_, 50 mL of 1 mM HAuCl_4_, 0.3 mL of 12.1 M HCl and 0.25 mL of 64 mM AA were subsequently injected into a mixture solution (50 mL) of CTAB (37 mM) and NaOL (4mM) and stirred for a brief time. Finally, 80 μL of seeds were injected into the growth solution. The resulting mixture was left undisturbed at 30 °C for 12 h. The final products were centrifuged twice (8000 rpm; 10 min) and stored at 100 $m$L of 80 mM CTAC solution for the further stages. The morphology and optical properties were characterized by the TEM images and UV-vis-NIR absorption spectrophotometer (**Figure S17**).

**Synthesis of chiral AuNRs (cAuNRs):** cAuNRs were synthesized according to the methodology developed in our laboratory. Firstly, 200 μL of 1 mM HAuCl_4_, 475 μL of 0.1 M AA, 20 μL of 10^-5^ M cysteine enantiomer (L-cys for L-cAuNRs and D-cys for D-cAuNRs) and 200 µL of AuNRs were successively added into 4 mL of 40 mM CTAC solution. After stirring for a while, the prepared solution was incubated for 90 min. The final products were collected by centrifugation (5000 rpm; 10 min) twice and stored in water for further use.

**Synthesis of chiral Pd-on-cAuNRs:** 200 μL of 10 mM Na_2_PdCl_4_, 2 mL of cAuNRs, and 400 μL of 100 mM AA were added into 20 mL of 10 mM CPC solution at 65 ℃. After stirring 2 min, the obtained solution was incubated for 30 min at 65 ℃. The final products were collected by centrifugation (5000 rpm; 5 min) twice and redispersed in water for further use.

**Polarized Photocatalysis:** To verify the circularly polarized photocatalytic performance of the chiral Pd-on-cAuNRs, three types of polarized light (LP: linearly polarized light; LCP: left circularly polarized light; RCP: right circularly polarized light) were tested. The laser beam (wavelength: 635 nm, initial power: 100 mW, beam shape: 10 × 10 mm^2^) was converted into LP, LCP and RCP with power of 20 mW light by passing it through a linear polarizer and a λ/4 polarizer (10 × 10 mm^2^ area, 2 nm thickness), respectively.

To evaluate the polarized photocatalysis on the POD-like nanozyme reaction, 1 mL of chiral Pd-on-cAuNRs and 200 µL of 0.416 mM TMB were sequentially added into 1.8 mL of acetate buffer solution (pH = 3.6). After stirring for a short time, 200 µL of 40 mM H_2_O_2_ were quickly added. Following that, a UV-Vis spectrum was recorded for 180 s. All experiments were performed at 35 ℃.

To calculate the steady-state enzymatic kinetic parameters of chiral Pd-on-cAuNRs nanozyme, various concentrations of either TMB or H_2_O_2_ were prepared in buffer solution always using 1 mL of nanozyme in each experiment. For the TMB studies, 200 µL of 0.2 mM, 0.3 mM, 0.416 mM, 0.5 mM, 0.8 mM and 1 mM TMB stock solutions were prepared in buffer while keeping 200 µL of 60 mM H_2_O_2_ as an unchanged parameter, respectively. In the case of H_2_O_2_ studies, 200 µL of 0.416 mM TMB was kept as a constant parameter, and 200 µL of 30 mM, 40 mM, 50 mM, 60 mM, 90 mM, 140 mM, 200 mM, 300 mM H_2_O_2_ stock solution were added, respectively. All experiments were conducted under different irradiation conditions (darkness, LP, RCP, LCP) and the temperature was stable at 30 ºC.

To further assess the polarized photocatalysis on 4-NP, a 0.1 mL of 100 mM 4-NP and 0.1 mL of 0.1 M NaBH_4_ were mixed with 1.8 mL water in a vial. Then, 600 μL of chiral Pd-on-cAuNRs were added into the solution. Afterwards, the solution was separately illuminated with a range of different polarized light. The UV-vis absorption spectra of the solution were recorded immediately at a specific time interval. All the experiments were performed under different lighting conditions (darkness, LP, RCP, LCP) and the temperature was stable at 30 ºC. As for the cycling experiments, the photocatalysts were cycled and collected through centrifugation from the reaction solution. Prior to centrifugation, 40 mM CTAB was added to the solution to improve the stability of the nanoparticles. After five cycles, the absorption feature of the solution at 652 nm exhibited no obvious difference, indicating that the nanozyme has good stability and reusability (**Figure S18**). In addition, after four cycles, the reduction rate of the absorption feature at 400 nm remained consistent, with values of 0.0434 min^-1^, 0.0428 min^-1^, 0.0402 min^-1^ and 0.0358 min^-1^. These results further demonstrate the excellent stability and reusability of the nanozyme (see **Figure S19** and **Figure S20**).

**Hot electron photocurrent measurements:** The hot electron photocurrent measurements were performed using the glassy carbon electrode. The photoanode was prepared by depositing the nanoparticles on the glassy carbon electrode substrate. The particle densities of D-Pd-on-cAuNRs and L-Pd-on-cAuNRs on the substrate were carefully adjusted to be appropriately equal. Na_2_SO_4_ electrolyte (0.1 M) were sandwiched between the substrate and a cover glass slide, and Pt wire was used as the counter electrode. A supercontinuum laser filtered at 633 nm was employed for illuminating the glassy carbon electrode. The photocurrent of the cell was measured by chopping the laser illumination. The laser spot size is about 28.26 mm^2^ and the power density is about 0.7 W cm^-2^. The hot electron photocurrent was recorded by a Keithley digital source meter.

**Instruments and Characterization:** Transmission electron microscope (TEM) images were recorded using a JEOL JEM-2100 microscope operating at 200 kV. Extinction spectra were obtained using a UV-1900 spectrophotometer. Circular dichroism (CD) spectra were acquired using a J-810 spectropolarimeter instrument (JASCO).


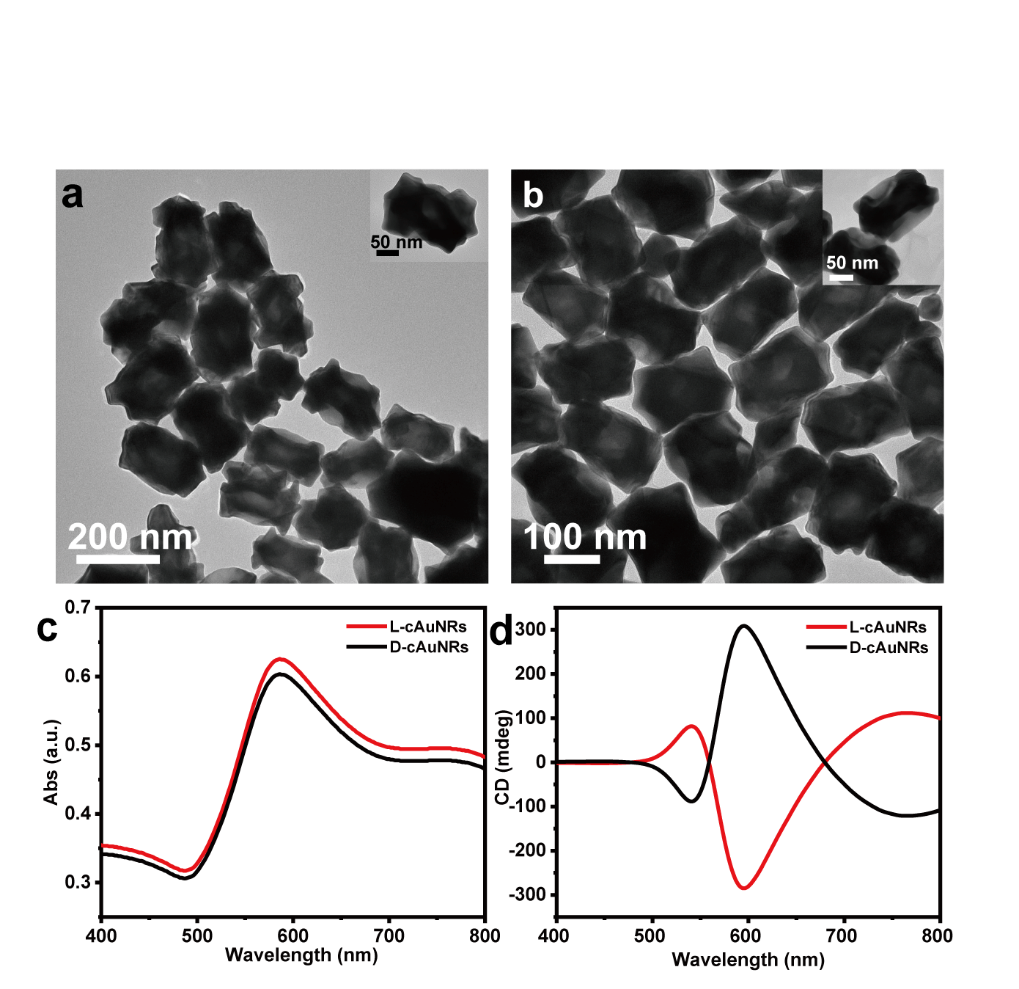


**Figure S1.** Characterization of cAuNRs. (a) TEM image of Lc-AuNRs, inset is a TEM image of single L-cAuNRs. (b) TEM image of D-cAuNRs; inset is a TEM image of single D-cAuNRs. (c) Vis-NIR absorption spectra of L-cAuNRs and D-cAuNRs. (d) The PCD spectrum of L-cAuNRs and D-cAuNRs.


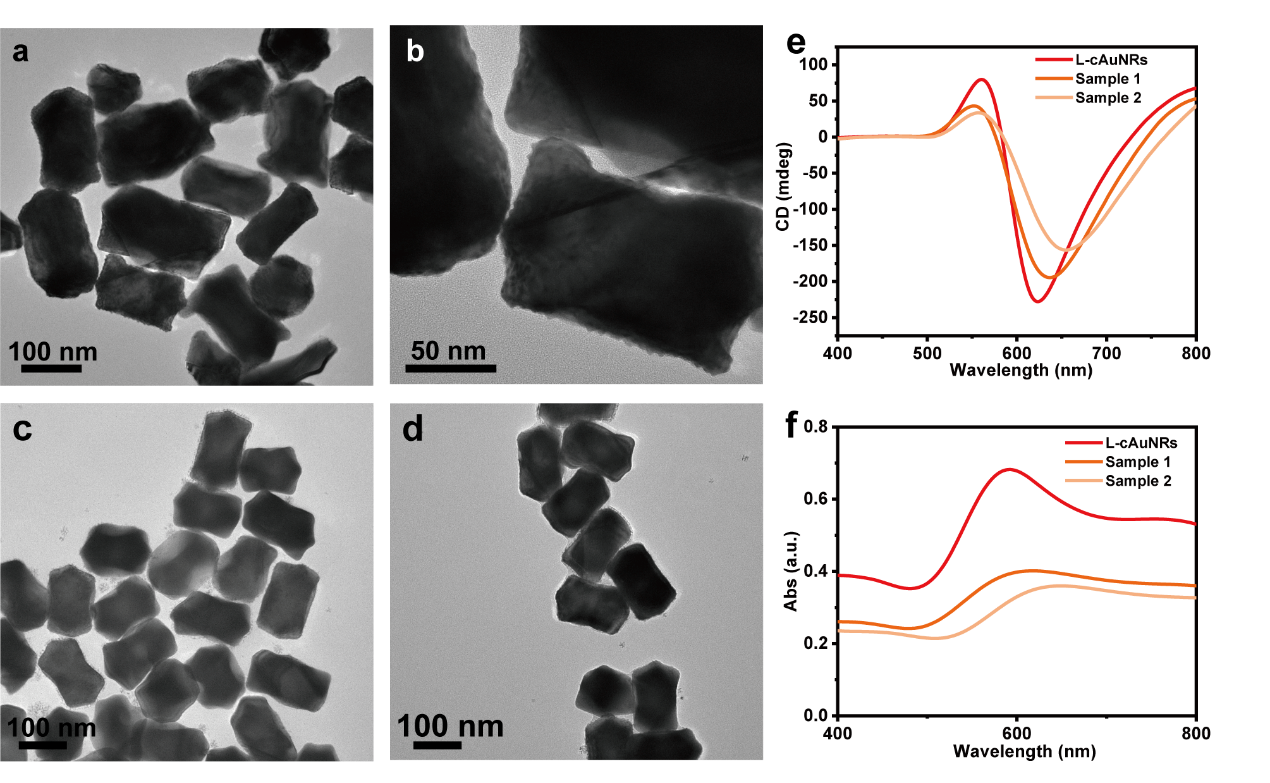


**Figure S2.** TEM images of sample 1 (a-b) and sample 2 (c-d). CD (e) and Vis-NIR absorption spectra (f) of sample 1 and sample 2. Sample 1: 200 μL of 10 mM Na_2_PdCl_4_, 200 μL of 100 mM AA; Sample 2: 400 μL of 10 mM Na_2_PdCl_4_, 400 μL of 100 mM AA.


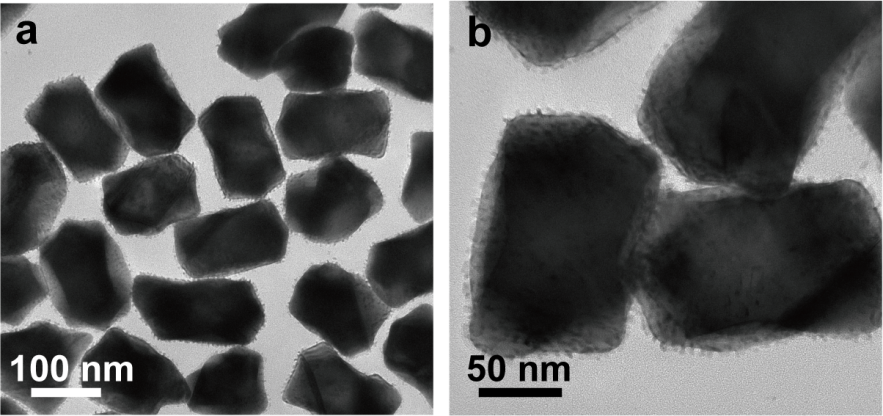


**Figure S3.** Low- (a) and high-magnification (b) TEM images of D-Pd-on-cAuNRs.


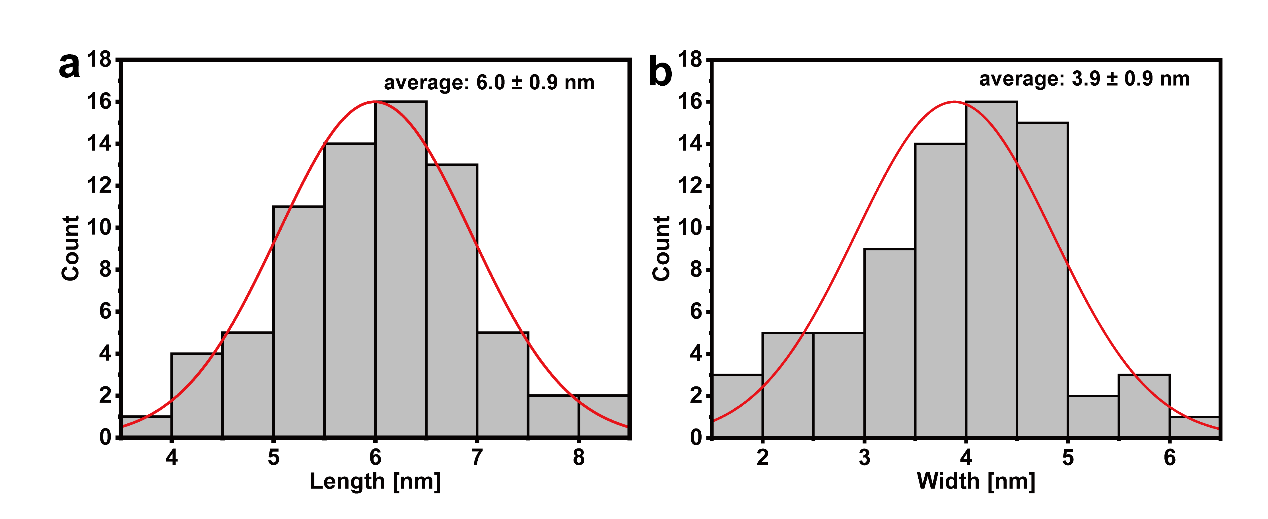


**Figure S4.** Statistical length (a) and width (b) of Pd nanoarrays.


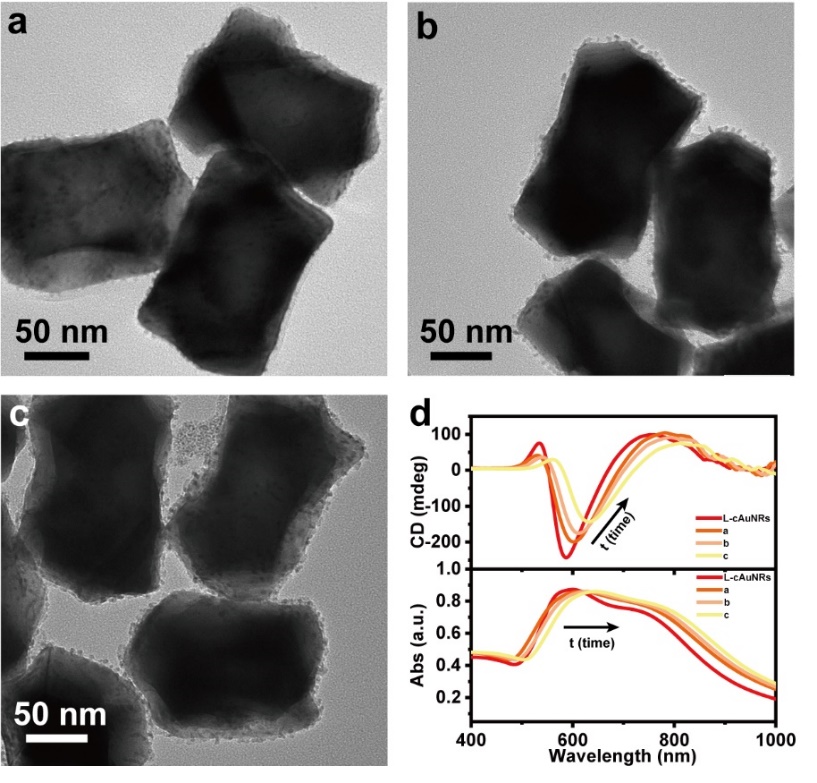


**Figure S5.** TEM images of products at (a) 0.5 min (b) 1 min and (c) 2 min, respectively. t=0 min is set after the injection of reducing agent AA. (d) Vis-NIR absorption spectra (top) and PCD spectra (bottom) of chiral Pd-on-cAuNRs collected at different growth stages.


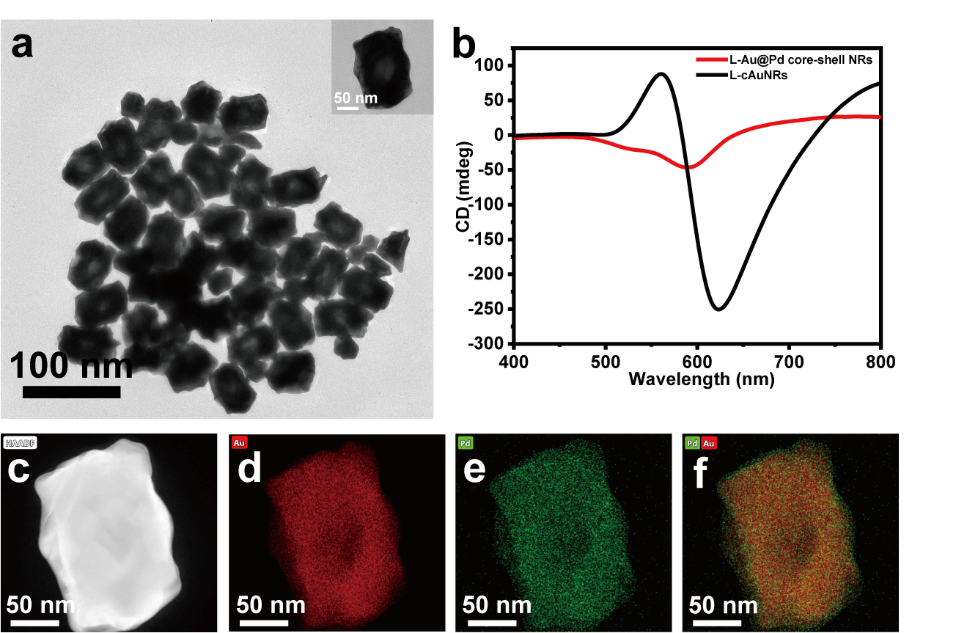


**Figure S6.** (a) Representative TEM image of L-Au@Pd core-shell NRs. Inset is a TEM image of single L-Au@Pd core-shell NRs. (b) The PCD spectrum of chiral Au@Pd core-shell NRs. (c) HAADF-STEM image of L-Au@Pd core-shell NRs. (d-f) EDX elemental mapping images for Au and Pd, respectively.


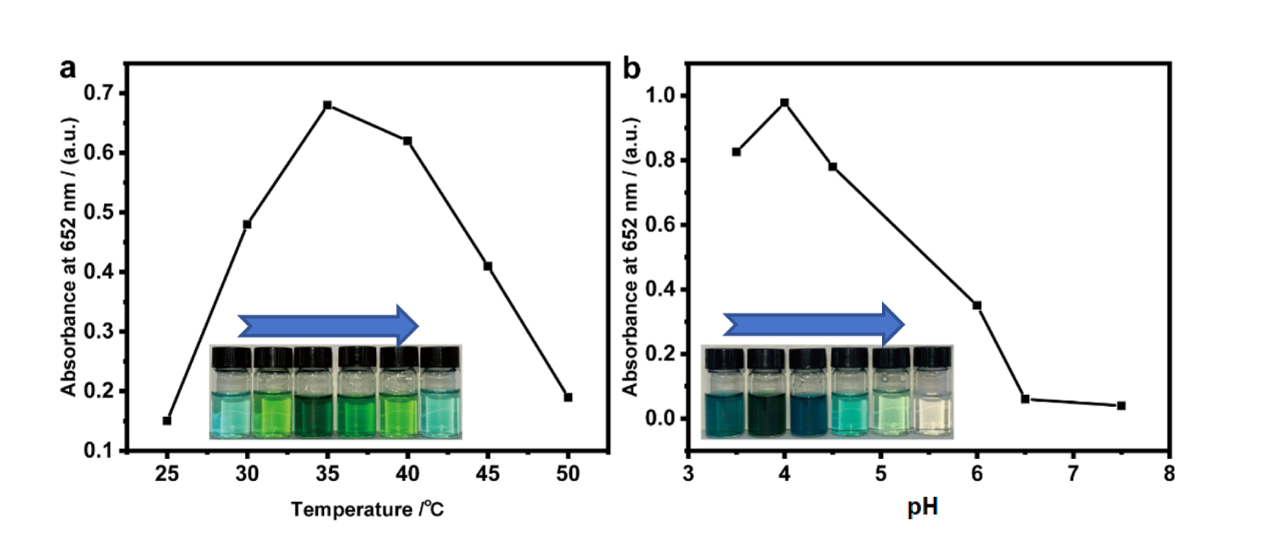


**Figure S7.** Effects of different experimental conditions on the Peroxidase-like activity of Pd-on-cAuNRs. The absorbance spectra and visual color changes of TMB in presence of different (a) temperature, (b) pH, respectively, with insets showing the corresponding photos of the reaction solutions.


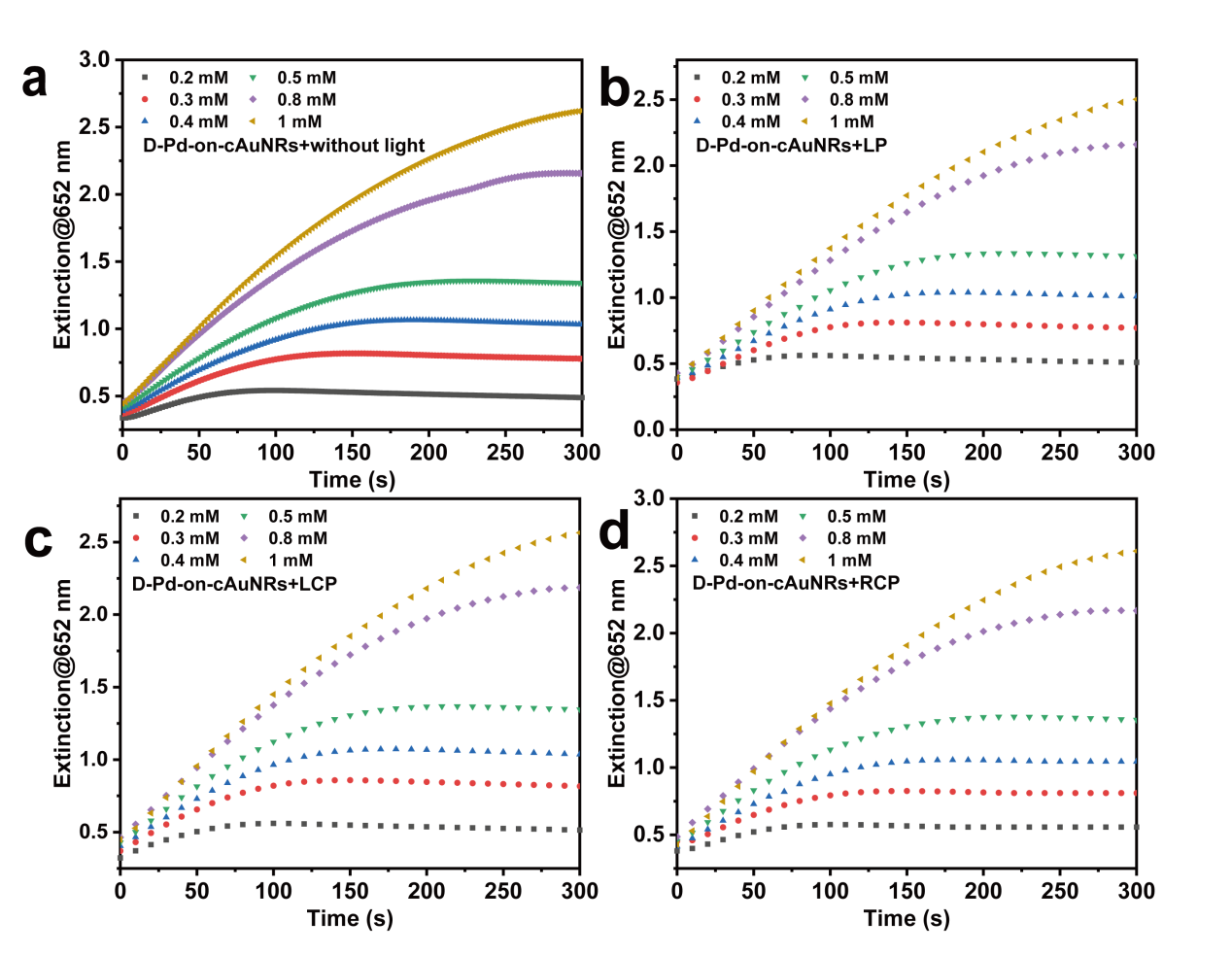


**Figure S8.** Time evolution of absorbance at 652 nm for different TMB concentrations, as indicated, using D-Pd-on-cAuNRs under dark conditions (a), LP (b), LCP (c) and RCP (d).


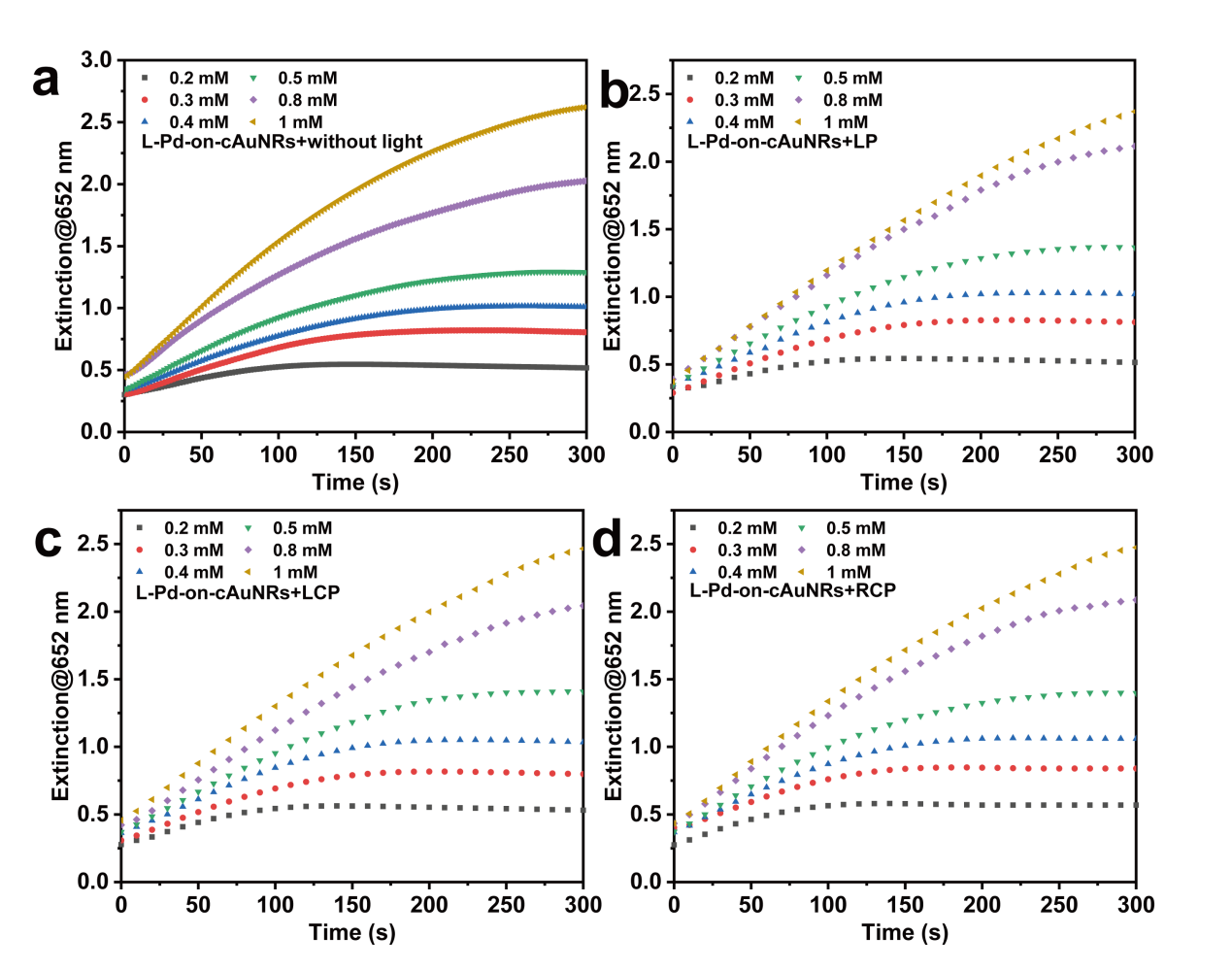


**Figure S9.** Time evolution of absorbance at 652 nm for different TMB concentrations, as indicated, with L-Pd-on-cAuNRs under dark conditions (a), LP (b), LCP (c) and RCP (d).


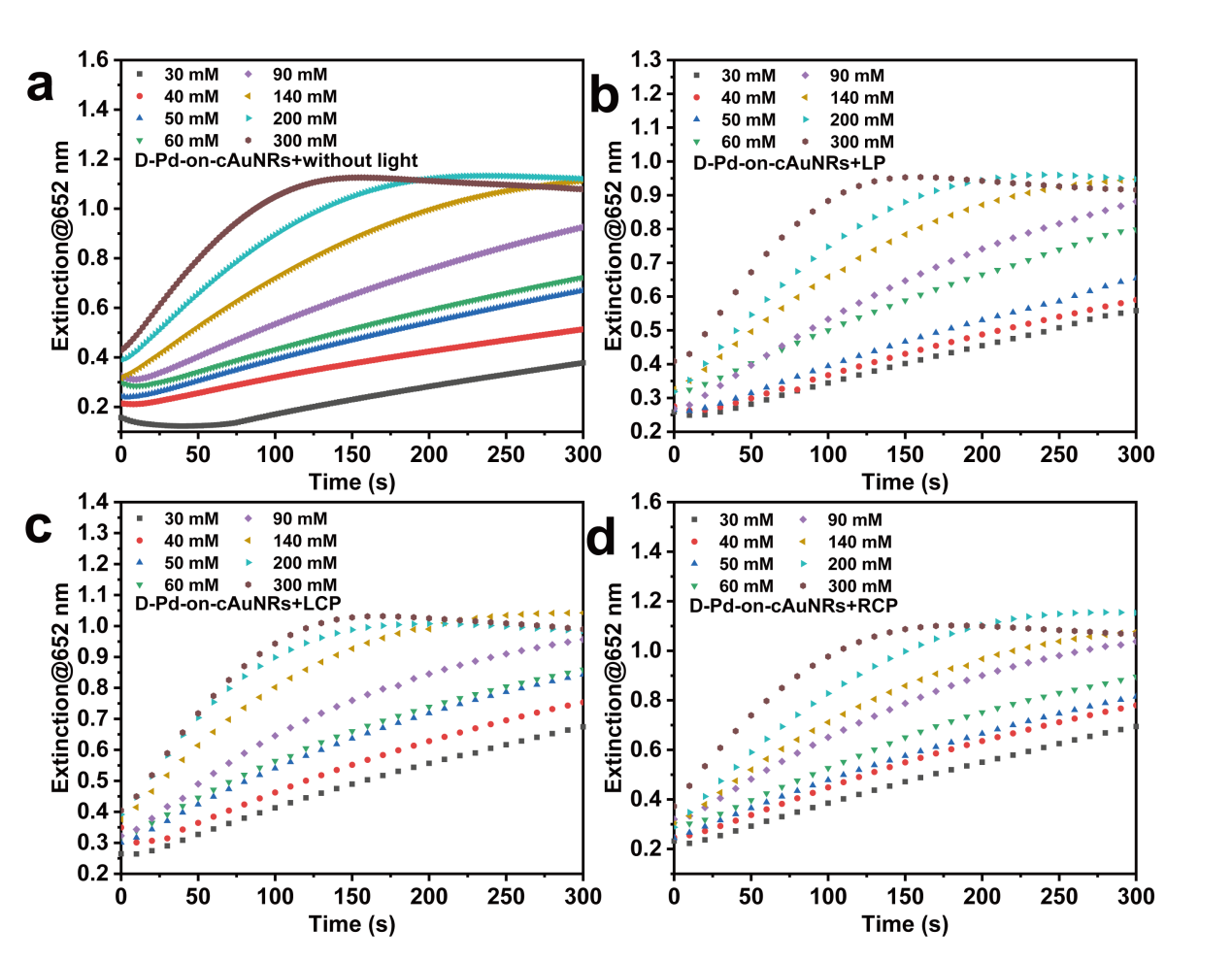


**Figure S10.** Time evolution of absorbance at 652 nm for different H_2_O_2_ concentrations, as indicated, employing D-Pd-on-cAuNRs under dark conditions (a), LP (b), LCP (c) and RCP (d).


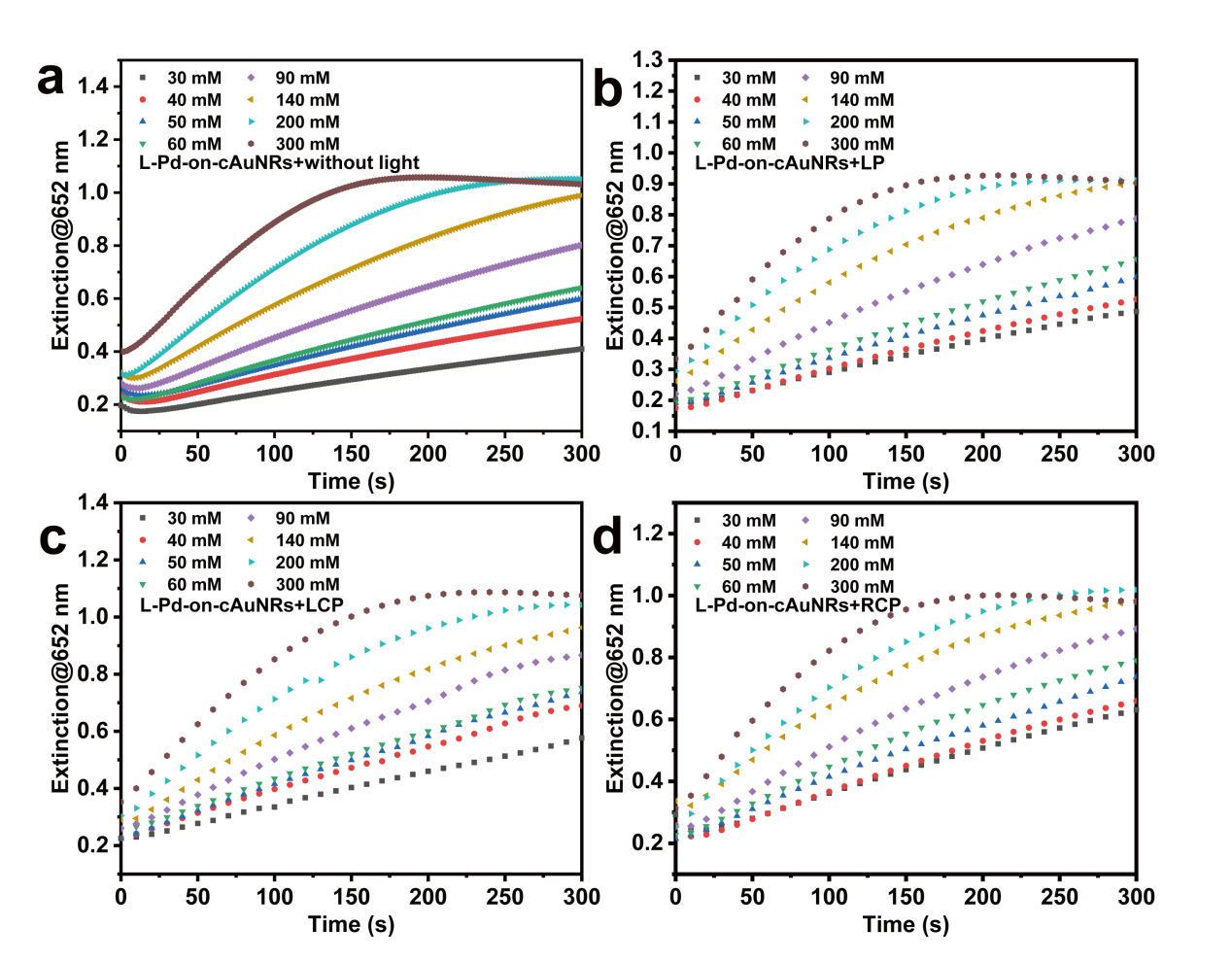


**Figure S11.** Time evolution of absorbance at 652 nm for different H_2_O_2_ concentrations, as indicated, with L-Pd-on-cAuNRs under dark conditions (a), LP (b), LCP (c) and RCP (d).


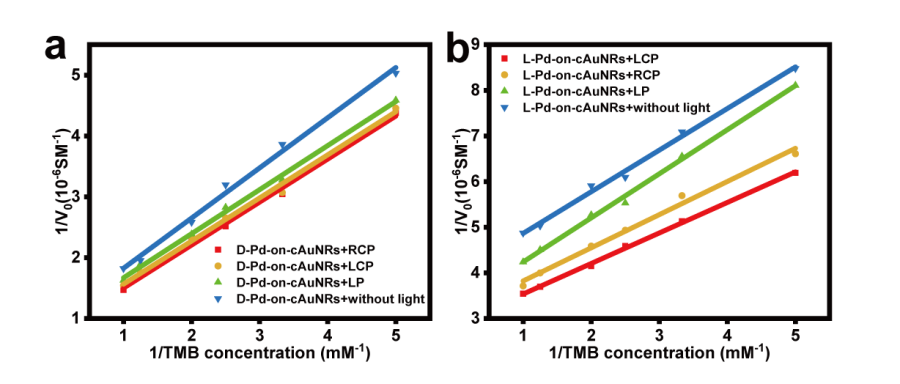


**Figure S12.** Lineweaver-Burk plots of D-Pd-on-cAuNRs (a) and L-Pd-on-cAuNRs (b) under different lighting conditions.


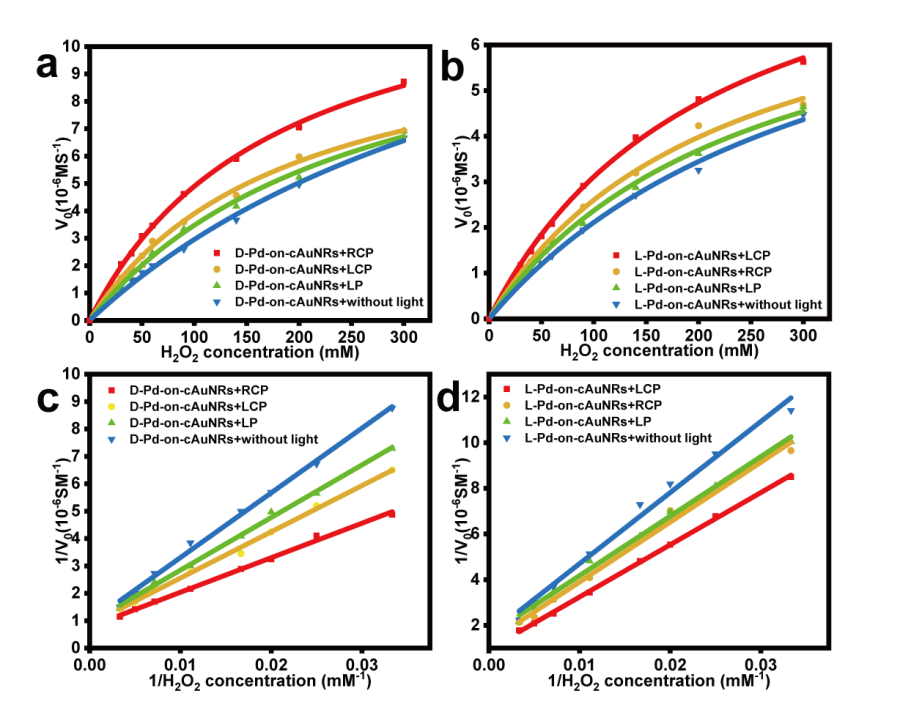


**Figure S13.** Michaelis-Menten curves and Lineweaver-Burk plots of D-Pd-on-cAuNRs (a, c) and L-Pd-on-cAuNRs (b, d) under different lighting conditions.


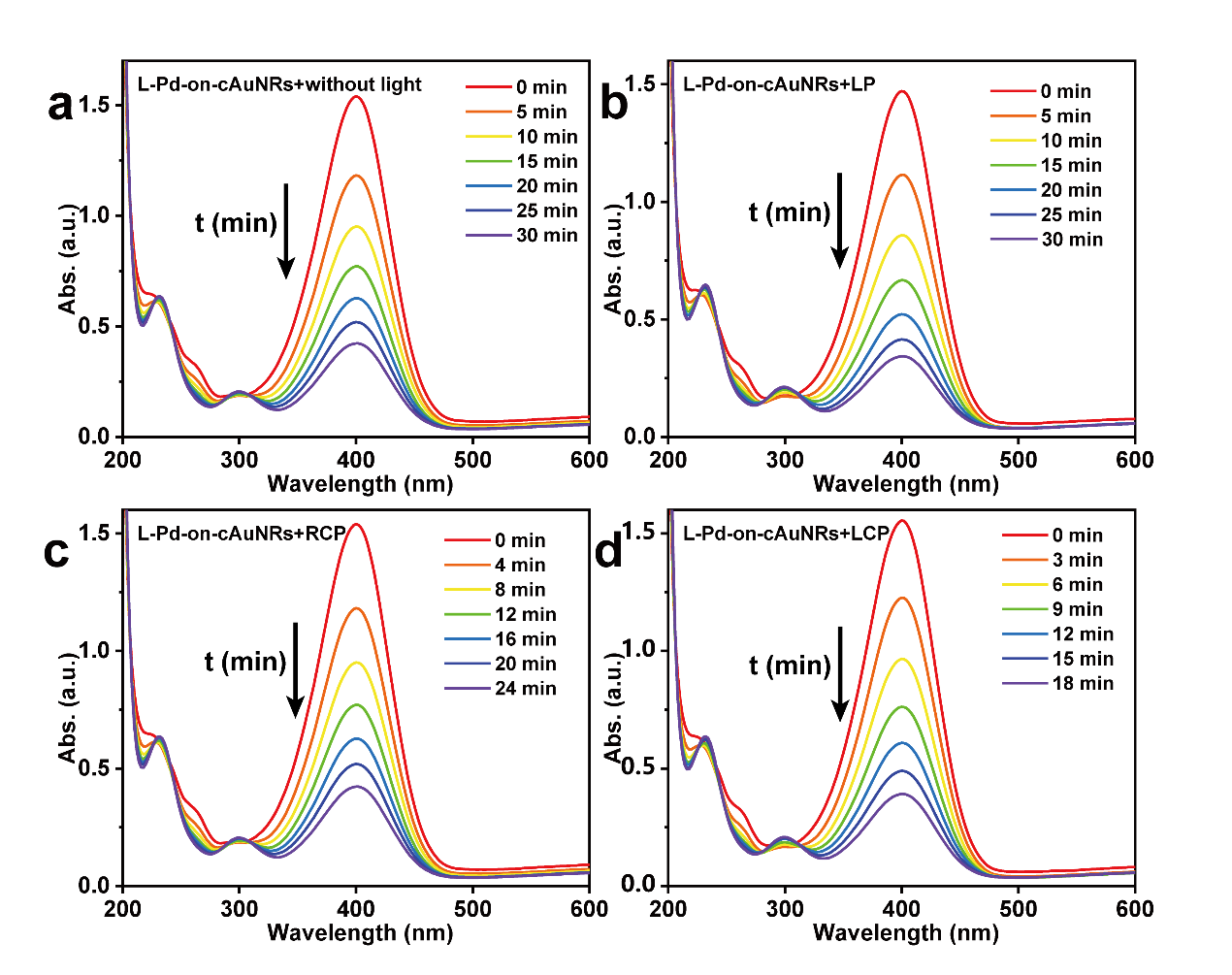


**Figure S14.** Time-dependent UV-vis absorption spectra of the catalytic reaction in the presence of L-Pd-on-cAuNRs under dark conditions (a), LP (b), RCP (c) and LCP (d).


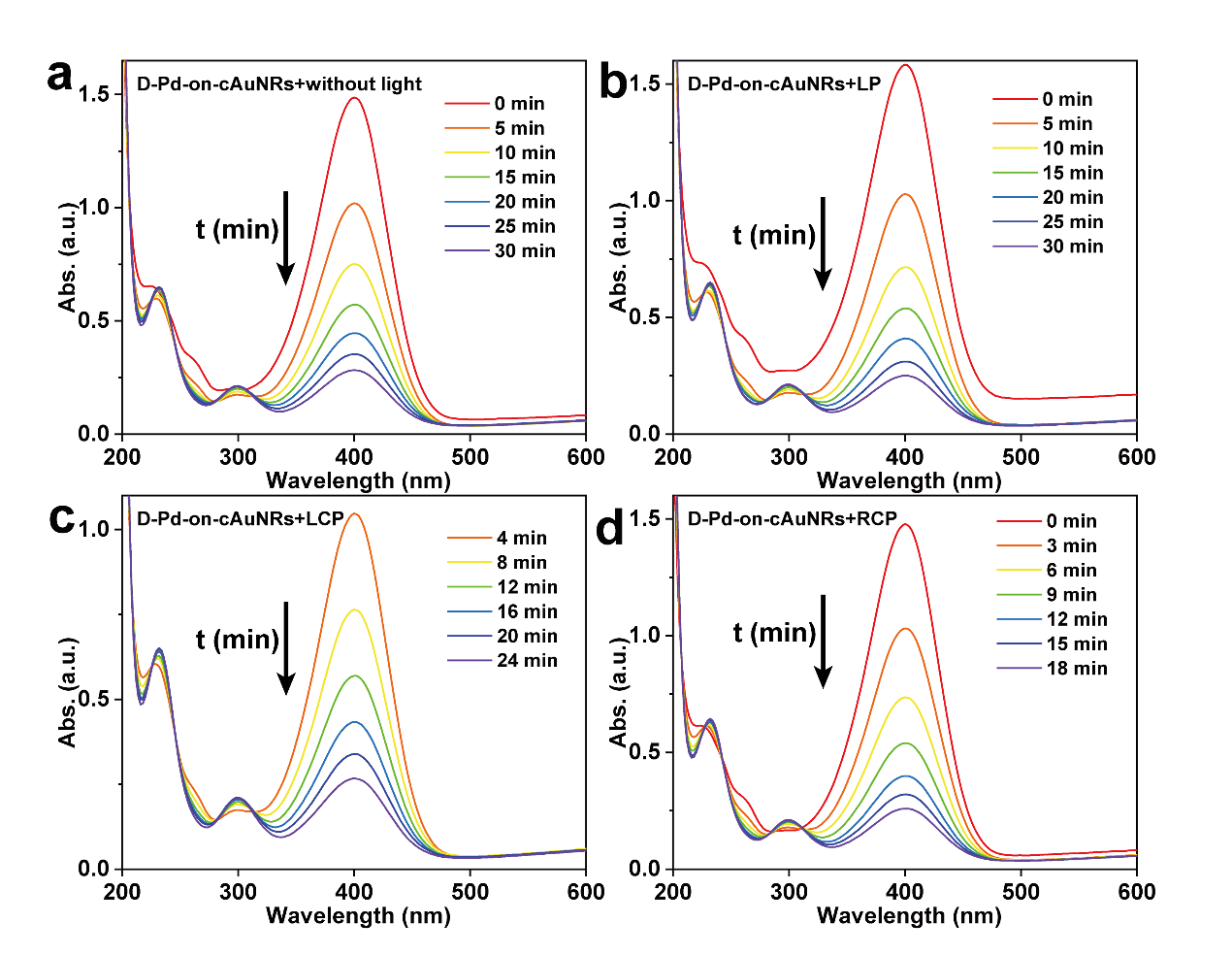


**Figure S15.** Time-dependent UV-Vis absorption spectra of the catalytic reaction in the presence of D-Pd-on-cAuNRs under dark conditions (a), LP (b), LCP (c) and RCP (d).


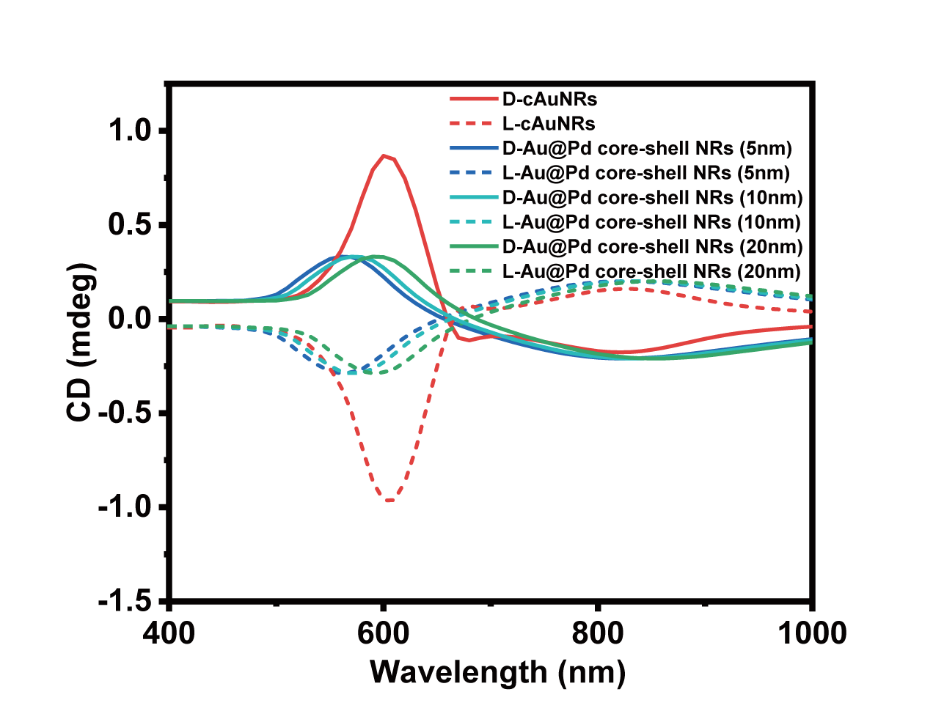


**Figure S16.** The PCD spectrum of chiral Au@Pd core-shell NRs with different thickness of core shell.


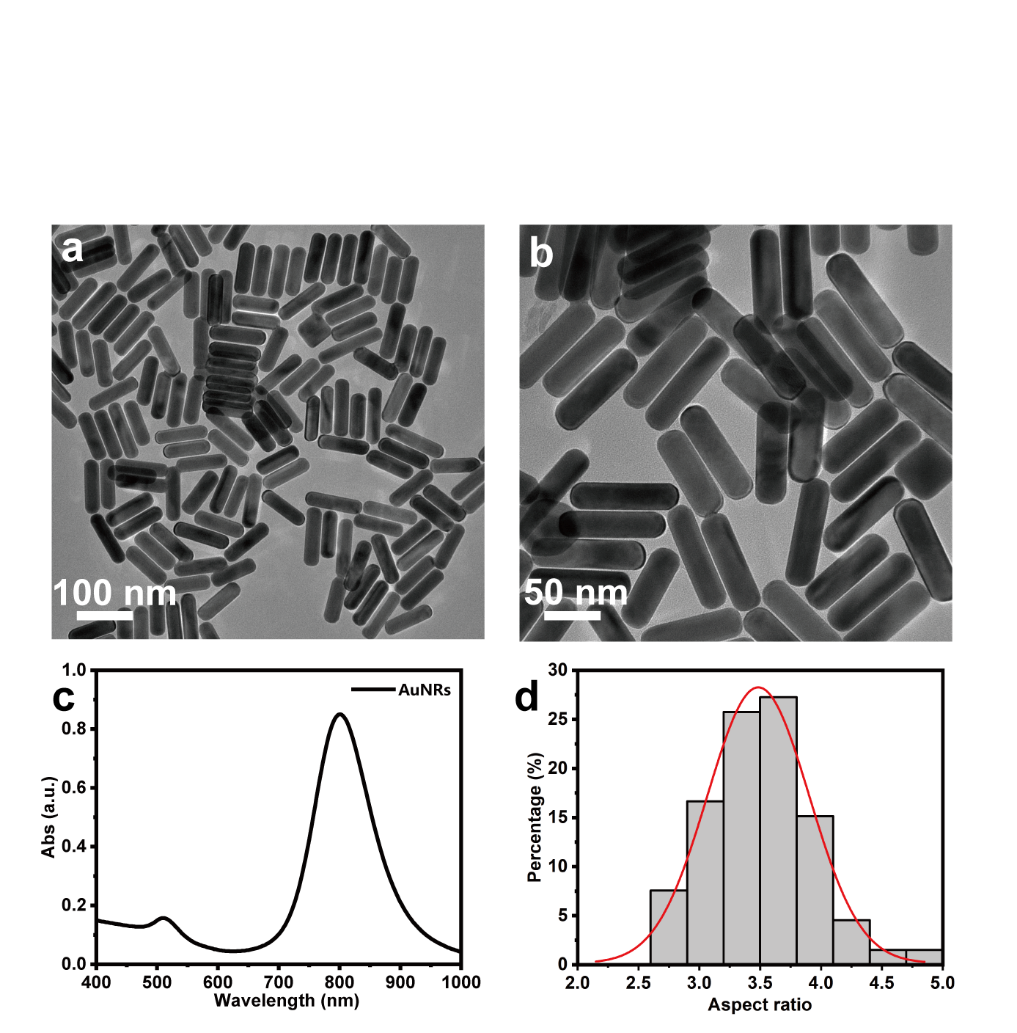


**Figure S17.** Characterization of AuNRs. (a, b) TEM images of AuNRs. (c) Vis-NIR spectra of AuNRs. (d) A statistical histogram of the aspect ratio of AuNRs.


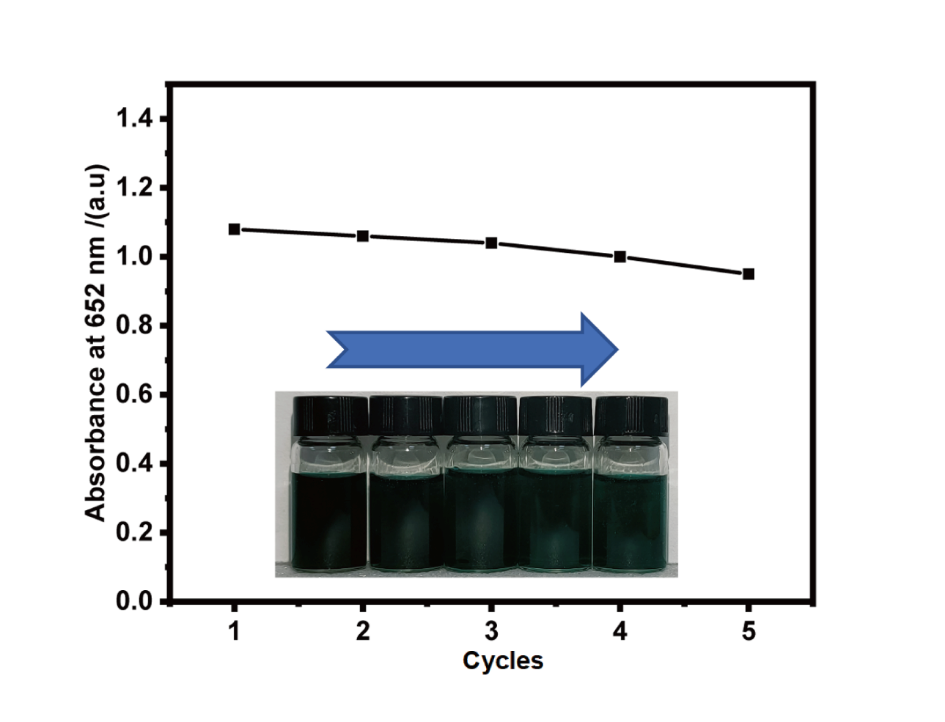


**Figure S18.** The intensity of absorbance at 652 nm were investigated for 5 cycles.


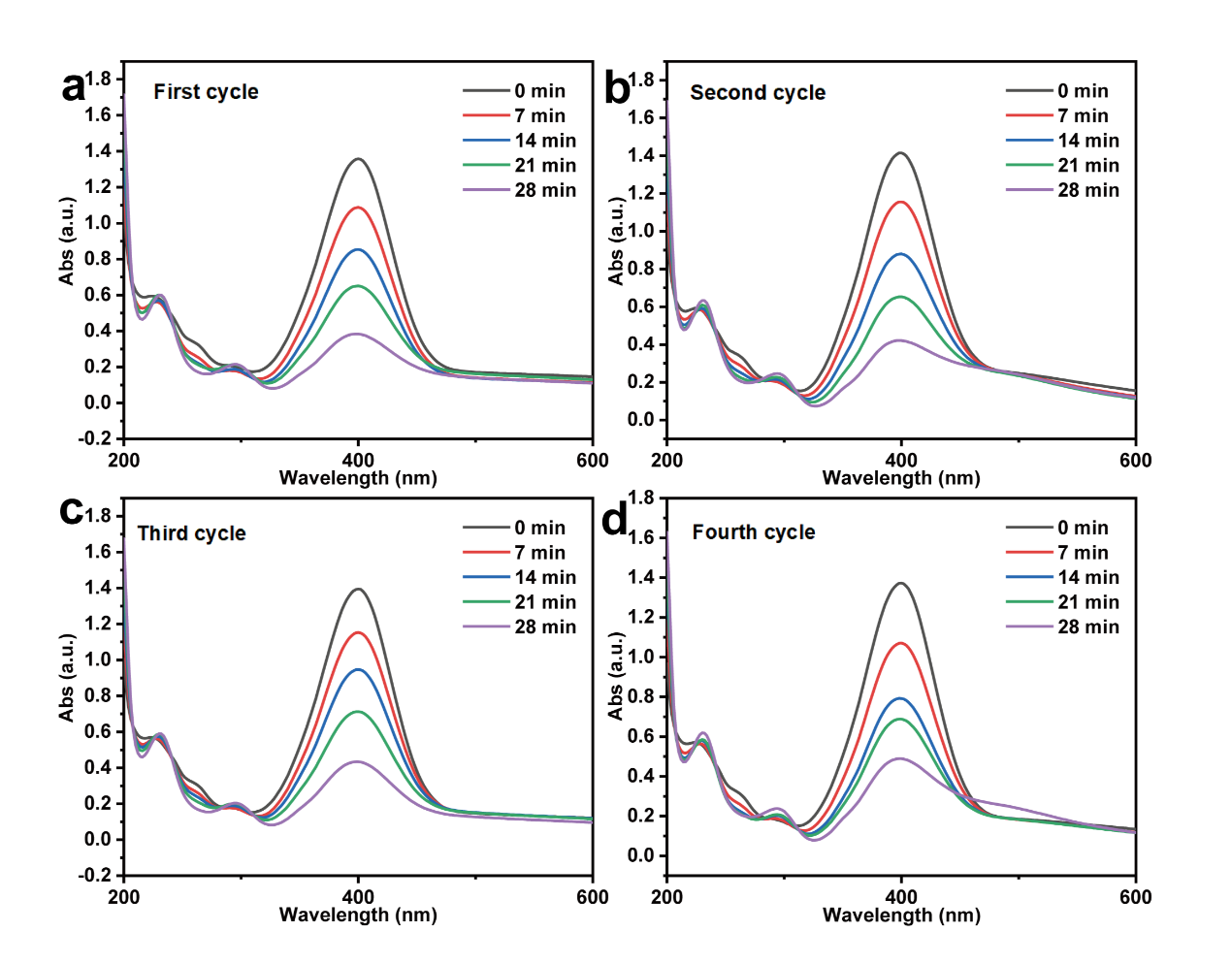


**Figure S19.** Time-dependent UV-vis absorption spectra of the catalytic reaction in the presence of D-Pd-on-cAuNRs after four cycles.


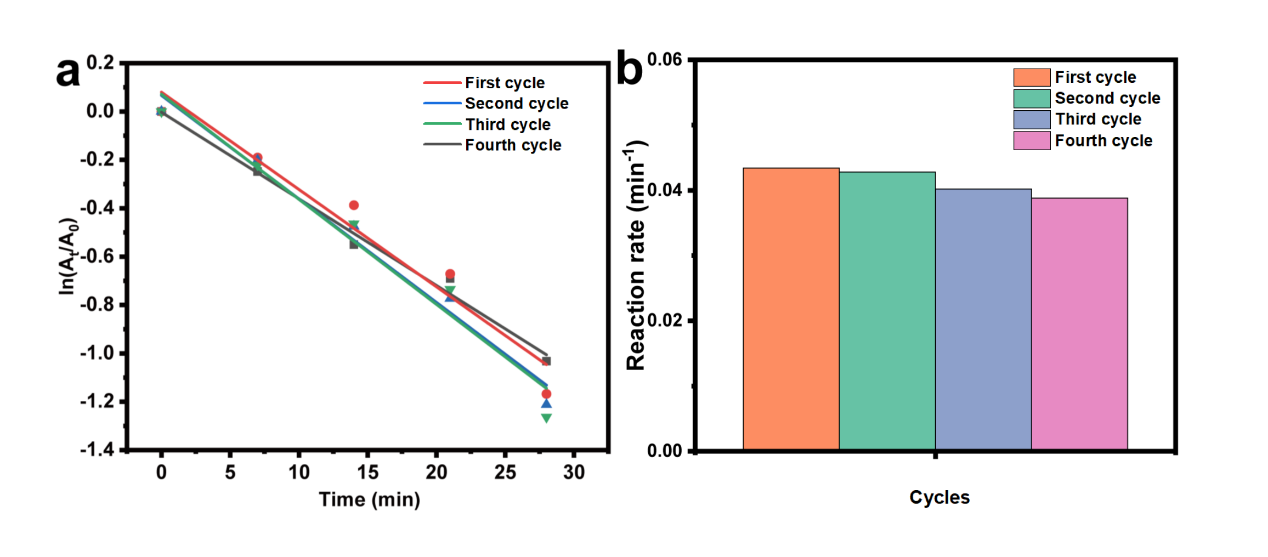


**Figure S20.** Plot of$Ln(A_{t}/A_{0})$ as a function of time for the reaction catalyzed by D-Pd-on-cAuNRs of the four cycles. (b) Histograms for the comparison of reaction rate by D-Pd-on-cAuNRs of the four cycles.

Reference

[1] G. C. Zheng, S. L. Jiao, W. Zhang, S. L. Wang, Q. H. Zhang, L. Gu, W. X. Ye, J. J. Li, X. C. Ren, Z. C. Zhang, K. Y. Wong, *Nano Res.* **2022**, *15*, 6574.
